# Guidelines for Perioperative Care for Liver Surgery: Enhanced Recovery After Surgery (ERAS) Society Recommendations 2022

**DOI:** 10.1007/s00268-022-06732-5

**Published:** 2022-10-30

**Authors:** Gaëtan-Romain Joliat, Kosuke Kobayashi, Kiyoshi Hasegawa, John-Edwin Thomson, Robert Padbury, Michael Scott, Raffaele Brustia, Olivier Scatton, Hop S. Tran Cao, Jean-Nicolas Vauthey, Selim Dincler, Pierre-Alain Clavien, Stephen J. Wigmore, Nicolas Demartines, Emmanuel Melloul

**Affiliations:** 1 Department of Visceral Surgery, Lausanne University Hospital CHUV, University of Lausanne (UNIL), Rue du Bugnon 46, 1011 Lausanne, Switzerland; 2 Hepato-Biliary-Pancreatic Surgery Division, Department of Surgery, Graduate School of Medicine, The University of Tokyo Hospital, Tokyo, Japan; 3 Department of Surgery, Flinders Medical Centre, Adelaide, Australia; 4 Department of Anesthesiology and Critical Care, University of Pennsylvania, Philadelphia, USA; 5 Leonard Davis Institute of Health Economics, University of Pennsylvania, Philadelphia, USA; 6 Department of Digestive and Hepato-Pancreato-Biliary Surgery, Assistance Publique-Hôpitaux de Paris (AP-HP), Hôpitaux Universitaires Henri Mondor, Créteil, France; 7 Department of Hepatobiliary Surgery and Liver Transplantation, Assistance Publique-Hôpitaux de Paris (AP-HP), Pitié-Salpêtrière Hospital, Paris, France; 8 Department of Surgical Oncology, The University of Texas MD Anderson Cancer Center, Houston, USA; 9 Department of Surgery, Swiss HPB Center, University of Zurich Hospital, Zurich, Switzerland; 10 Department of Surgery, University of Edinburgh, Edinburgh, UK

## Abstract

**Background:**

Enhanced Recovery After Surgery (ERAS) has been widely applied in liver surgery since the publication of the first ERAS guidelines in 2016. The aim of the present article was to update the ERAS guidelines in liver surgery using a modified Delphi method based on a systematic review of the literature.

**Methods:**

A systematic literature review was performed using MEDLINE/PubMed, Embase, and the Cochrane Library. A modified Delphi method including 15 international experts was used. Consensus was judged to be reached when >80% of the experts agreed on the recommended items. Recommendations were based on the Grading of Recommendations, Assessment, Development and Evaluations system.

**Results:**

A total of 7541 manuscripts were screened, and 240 articles were finally included. Twenty-five recommendation items were elaborated. All of them obtained consensus (>80% agreement) after 3 Delphi rounds. Nine items (36%) had a high level of evidence and 16 (64%) a strong recommendation grade. Compared to the first ERAS guidelines published, 3 novel items were introduced: prehabilitation in high-risk patients, preoperative biliary drainage in cholestatic liver, and preoperative smoking and alcohol cessation at least 4 weeks before hepatectomy.

**Conclusions:**

These guidelines based on the best available evidence allow standardization of the perioperative management of patients undergoing liver surgery. Specific studies on hepatectomy in cirrhotic patients following an ERAS program are still needed.

**Supplementary Information:**

The online version contains supplementary material available at 10.1007/s00268-022-06732-5.

## Introduction

Enhanced Recovery After Surgery (ERAS) is a multimodal and perioperative management pathway. ERAS offers to reduce the response to surgical stress and has been shown to decrease postoperative complications and length of stay (LoS) after several types of surgery [1, 2].

The first ERAS guidelines for liver surgery were published in 2016 [3]. Since then, several publications have shown that implementation of ERAS in liver surgery improves postoperative outcomes [4, 5]. Three recent meta-analyses showed that ERAS in liver surgery decreased postoperative complications, LoS, and costs [6–8]. In the first ERAS guidelines for liver surgery, 7 out of 23 recommendation items were based on non-liver surgery studies. The selected items described in the current updated recommendations are all issued from studies specifically on liver surgery.

The aim of the present systematic review was to update the guidelines for ERAS in liver surgery by reviewing the current literature and using a modified Delphi method to obtain an expert consensus.

## Material and methods

Creation of these guidelines followed the recommendations of the ERAS Society [9].

### Literature search and data selection

A systematic review was performed by searching MEDLINE/PubMed, Embase, and the Cochrane Library. For each specific ERAS item, a search query was created by two professional librarians. The specific queries for each item can be found in Supplementary files.

### Inclusion and exclusion criteria

Studies included in the previous guidelines and new studies published between January 1, 2010, and May 31, 2020, were considered. Eligible articles were meta-analyses, systematic reviews, reviews, expert consensus, randomized controlled trials (RCT), or prospective studies. Retrospective studies were considered only if better data were not available.

### Quality assessment and grade of recommendation

Levels of evidence of the recommendations were based on the Grading of Recommendations, Assessment, Development and Evaluations (GRADE) system, where levels were graded as low, moderate, or high [10]. The GRADE system includes RCT and observational studies. RCT were primarily defined as high level of evidence. Risk of bias, inconsistency, indirectness of evidence (different populations of interest or different outcomes), imprecision, and publication bias were then assessed, and, if present, level of evidence was downgraded by one or two steps. Observational studies were primarily defined as low level of evidence. The level of evidence was potentially upgraded by one or two levels based on the large magnitude of effect, the dose–response gradient, and the effect of residual confounding. The grades of recommendation, also based on the GRADE system, were defined as weak or strong [10]. Definition of the grade of recommendation considered the quality of evidence, the uncertainty of values and preferences, the balance between desirable and undesirable effect of alternative strategy of management, and the costs of intervention [10].

### Analyzed items

The items were chosen at the beginning of the study by the experts and were based on the previous guidelines and on the current available literature. Twenty-five recommendation items were elaborated.

### Modified Delphi method

An expert committee was chosen by the group responsible for the guidelines (Lausanne University Hospital group). The experts represent an international panel from Asia, America, and Europe. Twelve experts were asked by email whether they wanted to take part in the elaboration of these guidelines. All of them agreed. Each expert was then assigned 2–3 recommendation items to elaborate. After each expert wrote their individual parts, all items were put together by the editorial team. The Delphi rounds then started. For each round, the manuscript was sent individually to each expert to avoid influence of the results by reading other expert comments. Each expert was asked to comment and edit the manuscript using the Word Track Changes system. The editorial team edited the manuscript before every new round. Levels of evidence and grades of recommendation were modified for the next round only if the majority of experts agreed. A total of 3 web-based rounds was performed to reach the highest level of consensus. Consensus was determined to be reached if >80% agreement (i.e., 12/15 experts) was obtained for an item.

## Results

**Fig. 1:**
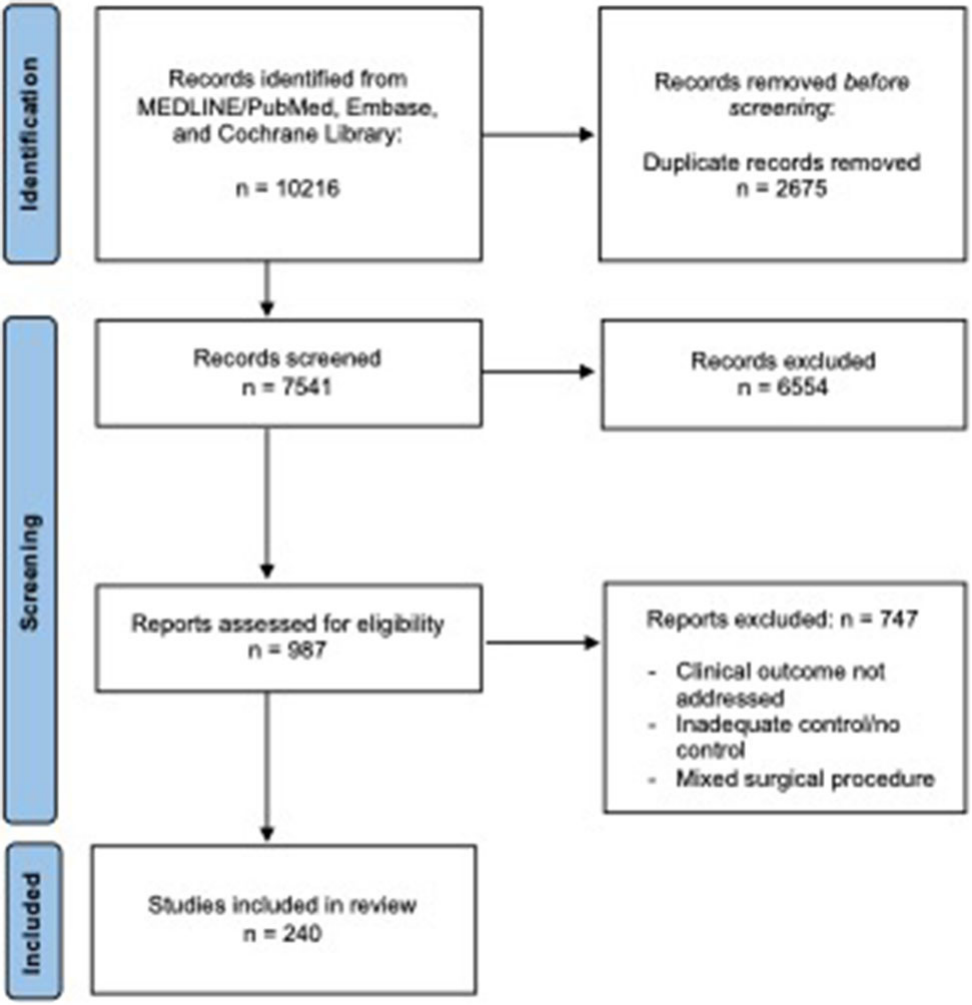
PRISMA flowchart of the systematic review of the literature

**Fig. 2:**
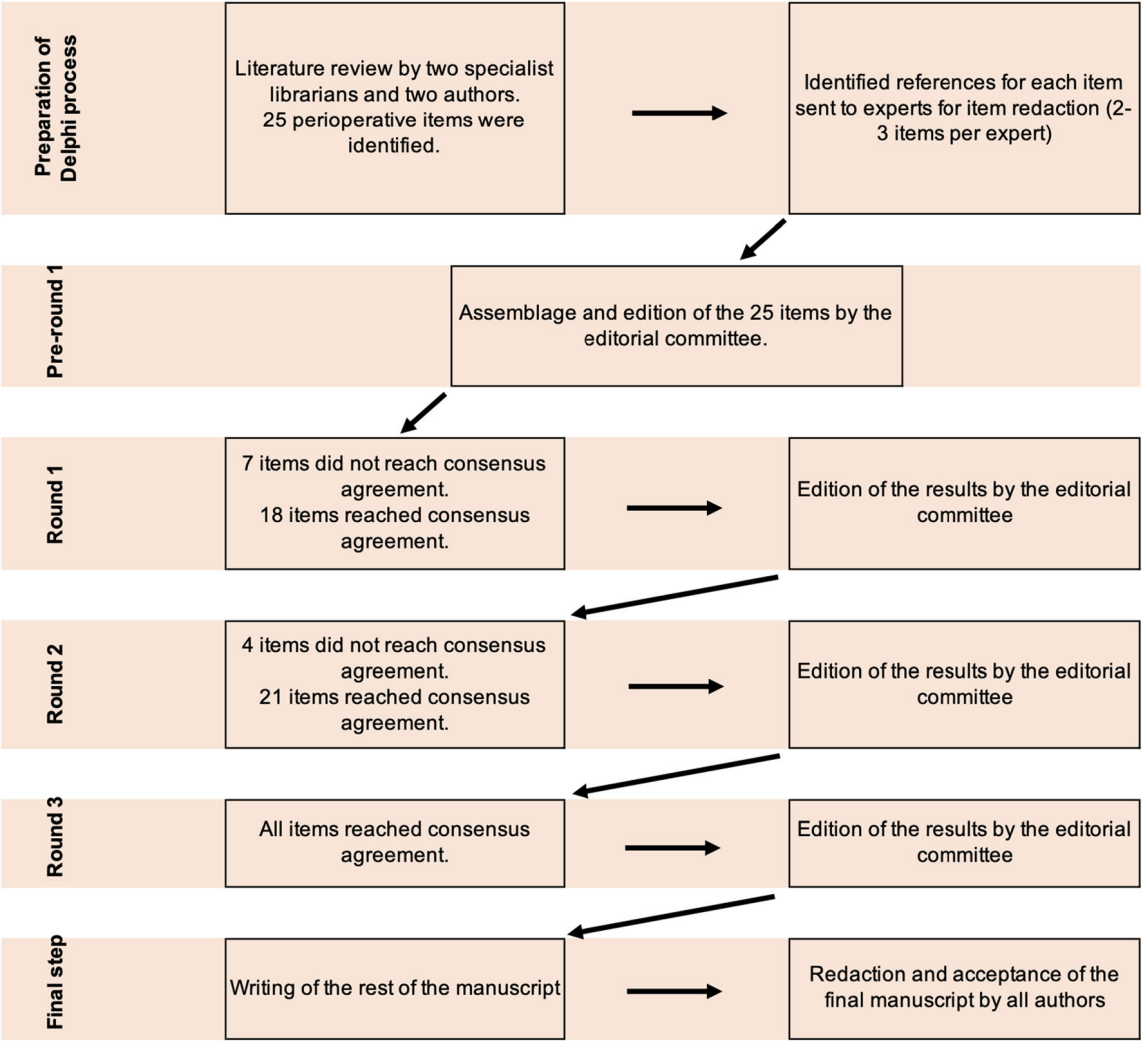
Process of the modified Delphi method for the development of the present consensus ERAS guidelines for perioperative care for liver surgery

The PRISMA flowchart of the study is summarized in Fig. 1, and the process of the modified Delphi method is depicted in Fig. 2. Table 1 shows the percentage of agreement for each round for items that did not reach consensus after round 1, and Table 2 summarizes the recommendations.

**Table 1:** Percentage of agreement (regarding summary, level of evidence, and grade of recommendation) after the different Delphi rounds for items that did not reach consensus (< 80% agreement) after the first round

	Round 1 (%)	Round 2 (%)	Round 3 (%)
Preoperative biliary drainage	67	86	93
Anti-thrombotic prophylaxis	73	93	100
Preoperative steroids administration	60	73	93
Prophylactic abdominal drainage	60	73	100
Delayed gastric emptying	73	100	100
Early and scheduled mobilization	73	75	100
Fluid management	73	75	100

**Table 2:** Summary of ERAS recommendations for liver surgery for each item, including the levels of evidence and the grades of recommendation

ERAS item	Summary	Evidence level	Grade of recommendation
1. Preoperative counseling	Patients should receive preoperative information and counseling regarding the upcoming liver surgery. Brochures and multimedia supports might help to improve the verbal counseling.	Low	Weak
2. Prehabilitation	Prehabilitation should be performed in high-risk patients (elderly, malnourished or overweight patients, smokers, or patients with psychological disorder) prior to liver surgery. Prehabilitation should be commenced 4–6 weeks before the operation depending upon the urgency of surgery. The content (physical exercises, dietary interventions, or anxiety reduction exercises) and duration of the prehabilitation program for liver surgery are not clearly established.	Moderate	Weak
3. Preoperative biliary drainage	Biliary drainage in cholestatic liver (>50 mmol/l) is recommended. For perihilar cholangiocarcinoma, percutaneous biliary drainage should be preferred to endoscopic biliary drainage. Surgery should ideally not be performed until bilirubin level drops below 50 mmol/l.	Moderate	Strong
4. Preoperative smoking and alcohol cessation	Preoperative smoking cessation should be counseled at least 4 weeks prior to hepatectomy. Alcohol cessation is recommended for heavy drinkers (> 24 g/day for women and >36 g/day for men) 4–8 weeks before surgery.	High	Strong
5. Preoperative nutrition	A nutritional assessment is necessary prior to all hepatic surgery. Malnourished patients (i.e., weight loss >10% or >5% over 3 months and reduced body mass index or a low fat-free mass index) should be optimized with enteral supplementation at least 7–14 days prior to surgery.	High	Strong
6. Perioperative oral immunonutrition	Due to the lack of evidence, the use of immunonutrition in hepatic surgery is not recommended yet.	Low	Weak
7. Preoperative fasting and preoperative carbohydrate load	Preoperative fasting of 2 h for liquids and 6 h for solids before anesthesia is safe and can be recommended.	Moderate	Strong
	Carbohydrate loading is recommended the evening before liver surgery and 2–4 h before induction of anesthesia. Preoperative carbohydrate loading is safe and improves perioperative insulin resistance, but it is not clear if it is associated with a reduction of length of stay in liver surgery.	Low	Weak
8. Pre-anesthetic medication	Long-acting anxiolytic drugs should be avoided, particularly in the elderly. Preoperative gabapentinoids and nonsteroidal anti-inflammatory drugs are not recommended. Preoperative acetaminophen should be dose-adjusted according to extent of resection. Preoperative hyoscine patches can be used in patients with high risk for postoperative nausea and vomiting but should be avoided in the elderly.	Moderate	Strong
9. Anti-thrombotic prophylaxis	Low molecular weight heparin or unfragmented heparin reduces the risk of thromboembolic events and should be routinely started postoperatively unless exceptional circumstances make this unsafe. Intermittent pneumatic compression devices should be used to further reduce this risk.	Moderate	Strong
10. Preoperative steroids administration	Steroid administration (methylprednisolone at a dose of 500 mg) is recommended. No recommendation can be formulated on diabetic patients undergoing liver surgery.	Moderate	Weak
11. Antimicrobial prophylaxis and skin preparation	Antibiotic prophylaxis (such as cefazolin) within 60 min before surgical incision is recommended, with no benefit extending it into the postoperative period. In case of complex liver surgery with biliary reconstruction, a targeted antibiotic pre-emptive regimen based on preoperative bile culture may be recommended but its duration is unknown.	Moderate	Weak
	Skin preparation with chlorhexidine-alcoholic solution is associated with a lower rate of surgical site infections, compared to povidone-iodine solution.	Moderate	Strong
12. Minimally invasive surgery	In trained teams and when clinically appropriate, laparoscopic liver resection is recommended since it reduces postoperative length of stay and complication rates.	Moderate	Strong
13. Epidural, postoperative intravenous, and postoperative per oral analgesia	For open liver surgery, thoracic epidural analgesia can provide excellent analgesia but has significant disadvantages. In fact, optimal postoperative management is key to avoid hypotension and mobility issues which can be detrimental to rapid recovery. Multimodal analgesia (including potential use of intrathecal opiates) is recommended.	High	Strong
	Regarding laparoscopic surgery, there is no need for regional anesthesia techniques, as multimodal analgesia combined with judicious intravenous opiates provides functional analgesia.	Low	Weak
14. Wound catheter and transversus abdominis plane (TAP) block	Continuous local anesthetic wound infiltration provides lower complication rates and overall equivalent analgesia to thoracic epidural analgesia. Local anesthetic transversus abdominis plane blockade as a supplement to standard analgesia improves pain control and reduces opiate usage.	High	Strong
15. Prophylactic nasogastric intubation	Prophylactic nasogastric intubation does not offer postoperative benefits and may in fact increase hospital length of stay. Routine use of prophylactic nasogastric intubation is not recommended.	High	Strong
16. Prophylactic abdominal drainage	The routine use of abdominal drain placement is not indicated for hepatectomy without biliary reconstruction. No recommendation can be made for hepatectomy with biliary reconstruction.	High	Strong
17. Preventing intraoperative hypothermia	Perioperative normothermia using multimodal temperature management (including circulating water garments or forced warm air) should be maintained during open and minimally invasive liver surgery.	Moderate	Strong
18. Postoperative artificial nutrition and early oral intake	Early oral intake with normal diet should be implemented after hepatectomy. Individualized need for artificial nutrition should be assessed for malnourished patients, patients with complications causing several days of fasting, and patients with liver cirrhosis. If artificial nutrition is considered, enteral administration should be preferred.	High	Strong
19. Postoperative glycemic control	Insulin therapy for maintenance of normoglycemia (<8.3 mmol/l) is recommended.	High	Strong
20. Prevention of delayed gastric emptying (DGE)	Use of an omental flap to cover the cut surface of the liver might reduce the risk of delayed gastric emptying after left-sided liver resection.	Low	Weak
21. Stimulation of bowel movement	Postoperative laxatives, gum chewing, herbal medicine, or decoction after hepatectomy might reduce the time to first flatus or stool but do not impact the morbidity rate. Current data do not permit the recommendation of the routine use of postoperative laxatives, gum chewing, herbal medicine, or decoction to stimulate bowel movement after liver surgery.	Moderate	Weak
22. Early and scheduled mobilization	Early mobilization (out of bed) after liver surgery should be established from the operative day until hospital discharge. No recommendation can be made regarding the optimal duration of mobilization.	Moderate	Strong
23. Postoperative nausea and vomiting (PONV) prophylaxis	A multimodal approach to postoperative nausea and vomiting should be used. Patients should receive postoperative nausea and vomiting prophylaxis with at least 2 antiemetic drugs such as dexamethasone and ondansetron.	High	Strong
24. Fluid management	Low central venous pressure (below 5 cm H_2_O) with close monitoring is recommended during hepatic transection. As maintenance fluid balanced crystalloid should be preferred over 0.9% saline or colloids. Goal-directed fluid therapy optimizes cardiac output and end-organ perfusion. This may be particularly beneficial after the intraoperative liver resection during a low central venous pressure state to restore tissue perfusion. Patients who have comorbidities and reduced cardiac function may benefit most.	High	Strong
25. Monitoring/Audit	Substantial literature exists supporting that audit and feedback improve outcomes in health care and surgery. Regular audit and feedback should be implemented and performed in liver surgery to monitor and improve postoperative outcomes and compliance to the ERAS program.	Moderate	Strong

### Preoperative counseling

No RCT assessing preoperative counseling specifically in liver surgery exist. A recent RCT (PEDUCAT trial) compared a 1-h preoperative counseling and information seminar associated with brochure to brochure only with standard management (control group) in major abdominal surgery, including 25 liver resections [11]. No difference in complications or mortality was observed between both groups except for hospital falls, which were more frequent in the control group. Moreover, no difference in patient satisfaction was found. The preoperative information seminar was nevertheless beneficial for the training of patients and nursing staff. In one study, a mobile application for hepatopancreatobiliary (HPB) surgery within an ERAS pathway containing preoperative information was developed and was found to be feasible [12]. A systematic review on preoperative information before elective surgery did not show any differences in terms of perioperative anxiety and postoperative outcomes between specific formats and timings [13].

**Summary and recommendation:** Patients should receive preoperative information and counseling regarding the upcoming liver surgery. Brochures and multimedia supports might help to improve the verbal counseling.

**Evidence level:** Low.

**Grade of recommendation:** Weak.

### Prehabilitation

Two recent systematic reviews (including 419 and 1377 patients) focusing on prehabilitation for liver surgery have been published [14, 15]. Both found no difference in terms of postoperative complications and LoS. Only a trend toward less postoperative complications and shorter LoS was found in the pooled analysis of the systematic review by Dewulf et al*.* [15]. Both reviews underlined that several included studies were underpowered and that standardized outcome measures should be defined in future analyses. A narrative review on prehabilitation for patients with steatosis suggested that the 4–6-week period before the operation could be used for prehabilitation including dietary intervention to decrease intrahepatic fat and improve postoperative outcomes [16]. Another further narrative review on older patients recommended a focus on high-risk patients who could benefit from prehabilitation [17]. Aging is often associated with sarcopenia and malnutrition, rendering these patients more at risk of a complication [17]. Frail patients might therefore benefit the most from prehabilitation [17]. In their review, Walcott-Sapp and Billingsley [18] recommended that candidates for major liver resection should have their nutritional and functional status evaluated preoperatively and improved if necessary. Two RCT on prehabilitation in liver surgery have been performed [19, 20]. One study, including a total of 51 patients, found that postoperative complications were similar but that serum insulin levels were decreased and anaerobic threshold increased in the prehabilitation group [19]. The other RCT of 35 patients found an improvement in cardiopulmonary testing and quality of life in patients who had prehabilitation [20]. Two other studies specific to liver surgery (one prospective study and one propensity score matching study) found improved outcomes in the prehabilitation group (decreased complications and LoS) [21, 22]. Nine systematic reviews and meta-analyses on major abdominal surgery found globally that postoperative complications were reduced in patients having prehabilitation but heterogeneity of the included studies was high and quality of the evidence low [23–31].

**Summary and recommendation:** Prehabilitation should be performed in high-risk patients (elderly, malnourished or overweight patients, smokers, or patients with psychological disorder) prior to liver surgery. Prehabilitation should be commenced 4–6 weeks before the operation depending upon the urgency of surgery. The content (physical exercises, dietary interventions, or anxiety reduction exercises) and duration of the prehabilitation program for liver surgery are not clearly established.

**Evidence level:** Moderate.

**Grade of recommendation:** Weak.

### Preoperative biliary drainage (PBD)

The results of 2 meta-analyses showed that the mortality rate was similar between patients with or without PBD for perihilar cholangiocarcinoma, but that PBD increased the incidence of complications such as pancreatitis, cholangitis, and surgical site infection (SSI) [32, 33]. In the meta-analysis by Moole et al. [34], PBD was associated with fewer overall major adverse events than surgery itself, especially in patients undergoing endoscopic PBD. Moreover, PBD has been proven beneficial in the presence of cholangitis, severe malnutrition, and coagulation abnormalities [35]. Most reports have described PBD on the future liver remnant side. Prolonged preoperative jaundice was associated with increased postoperative morbidity and mortality after hepatic resection because of severe cholestatic liver dysfunction [36]. Regarding hilar cholangiocarcinoma, an expert consensus statement (American Hepato-Pancreato-Biliary Association-sponsored consensus meeting) recommended PBD in patients with cholangitis, hyperbilirubinemia-induced malnutrition, hepatic insufficiency, or renal insufficiency and in patients undergoing preoperative chemotherapy or portal vein embolization [37].

Endoscopic biliary drainage (EBD) and percutaneous biliary drainage (PTBD) for hilar tumors are the 2 main strategies of PBD. According to different meta-analyses, PTBD is associated with a lower rate of complications such as pancreatitis and cholangitis than EBD, and PTBD has a higher therapeutic success rate than EBD [38–40]. Conversely, a meta-analysis by Wang et al. [41] showed that the incidence of seeding metastasis was significantly higher in the PTBD than EBD group, and EBD was superior to PTBD in terms of overall survival in patients with resectable perihilar cholangiocarcinoma.

Neither the timing of surgery nor the duration of PBD has been defined. Most institutions define these parameters based on the serum bilirubin concentration, which also shows variance. Some centers recommended PBD duration until the bilirubin level is <2 to 3 mg/dl (about 30–50 mmol/l) [37]. Only one study has assessed the optimal interval between PBD and liver resection [42]. Son et al. [42] classified patients into either a long-term (≥2 weeks) or a short-term (<2 weeks) group. They showed that PBD <2 weeks before surgery was associated with significantly fewer PBD-related complications after resection.

**Summary and recommendation:** Biliary drainage in cholestatic liver (>50 mmol/l) is recommended. For perihilar cholangiocarcinoma, percutaneous biliary drainage should be preferred to endoscopic biliary drainage. Surgery should ideally not be performed until bilirubin level drops below 50 mmol/l.

**Evidence level:** Moderate.

**Grade of recommendation:** Strong.

### Preoperative smoking and alcohol cessation

Smoking is a risk factor for overall complications, SSI, pulmonary complications, neurological complications, and admission to the intensive care unit after surgery [43]. Lv et al. [44] showed in a retrospective study of 425 patients that smoking was an independent risk factor for liver-related and infectious complications after hepatectomy in patients with hepatocellular carcinoma (HCC). No prospective study specific to smoking cessation and liver surgery has been published yet. Two older RCT found a benefit of preoperative smoking cessation in terms of complications before various types of operations [45, 46]. A systematic review of 25 articles published in 2012 confirmed that smoking cessation at least 4 weeks before surgery reduced the risk of respiratory and wound-associated complications [47]. Conversely, smoking cessation less than 4 weeks prior surgery did not improve postoperative outcomes. A Cochrane systematic review published in 2014 including 13 RCT found that an intensive intervention for smoking cessation prior to surgery reduced postoperative complications compared to no intervention (risk ratio 0.42) [48]. Two retrospective studies also suggested that smoking was a risk factor for higher recurrence and liver-specific mortality after hepatectomy for HCC [49, 50].

In a systematic review with meta-analysis including 55 studies, alcohol was found to be an independent risk factor for overall, infectious, and respiratory complications after surgery [51]. Nevertheless, low-to-moderate alcohol consumption was not associated with postoperative morbidity but data remained scarce [51]. In liver surgery, alcoholic hepatitis is a risk factor for postoperative complications [52]. Alcohol consumption should therefore be reduced and ideally stopped in the perioperative period.

**Summary and recommendation:** Preoperative smoking cessation should be counseled at least 4 weeks prior to hepatectomy. Alcohol cessation is recommended for heavy drinkers (>24 g/day for women or >36 g/day for men) 4–8 weeks before surgery.

**Evidence level:** Smoking: high, alcohol: high.

**Grade of recommendation:** Smoking: strong, alcohol: strong.

### Preoperative nutrition

Prior to hepatic surgery, a nutritional risk screening is required to determine patients at higher risk of postoperative complications. Numerous nutritional screening tools have been validated [53]. Multiple meta-analyses have prognosticated nutritional scoring systems utilizing a combination of serum albumin and lymphocyte count (prognostic nutritional index) [54, 55] and, in addition, serum cholesterol (controlling nutritional status score) [56] in patients undergoing hepatectomy for HCC.

Delaying surgery to optimize preoperative malnutrition (body mass index, BMI <18.5 kg/m^2^) and disease-related malnutrition (weight loss >10% or >5% over 3 months and reduced BMI or a low fat-free mass index) is necessary [57]. A number of adverse outcomes have been associated with poor perioperative nutrition, including septic complications [58, 59]. Perioperative enteral nutritional therapy should be utilized for a period of 7–14 days in such patients, preferably in an outpatient setting [57]. Parenteral nutrition should only be considered in patients where requirements cannot be met by enteral nutrition alone [57]. A single RCT has demonstrated that intraoperative blood loss can be minimized in obese patients undergoing hepatectomy by introducing a low-fat, low-calorie diet one week prior to surgery [60].

**Summary and recommendation**: A nutritional assessment is necessary prior to all hepatic surgery. Malnourished patients (i.e., weight loss >10% or >5% over 3 months and reduced body mass index or a low fat-free mass index) should be optimized with enteral supplementation at least 7–14 days prior to surgery.

**Evidence level**: High.

**Grade of recommendation**: Strong.

### Perioperative oral immunonutrition

Immunomodulation through the use of branched-chain amino acids (BCAA), L-arginine, omega-3 fatty acids, and nucleotides has been reported to control inflammation, prevent immunosuppression, and thus reduce postoperative sepsis [59, 61].

The reduction of inflammation and improvement in hepatic function as a result of immunonutrition may improve outcomes. Omega-3 fatty acid administration has been associated with a reduction in infections and improved liver functions in patients undergoing hepatic resection [62]. Yang et al. [63] suggested that omega-3 fatty acid administration in rats undergoing large hepatic resection reduced hepatic fibrosis and improved hepatic regeneration. Akbari et al. [64] failed to demonstrate this benefit in rats, although the hepatic resections were significantly smaller. Beppu et al. [65] concluded that BCAA supplementation in humans was beneficial in hepatic regeneration after portal vein embolization.

A few systematic reviews suggested promising results. Decreased postoperative complications, improved nutritional state, and shortened hospitalization have all been demonstrated [59, 66]. Numerous biochemical parameters (alanine aminotransferase, aspartate aminotransferase, white cell count and pre-albumin) have been used and have demonstrated a benefit of omega-3 fatty acids in hepatic surgery [67]. None of these beneficial aspects have shown any improvement in postoperative mortality [59, 66–68]. McKay et al. [59] noted that the majority of RCT in their systematic review were of poor quality. More recent RCT failed to demonstrate a benefit for immunonutrition in elective hepatic surgery [69, 70]**.**

Ichikawa et al. [71] demonstrated an oncological benefit for the use of BCAA. Tumor recurrence (at 30 months) as well as alpha-fetoprotein (at 36 months) was significantly reduced as a result of BCAA use; however, overall mortality was unaffected.

The European Society for Clinical Nutrition and Metabolism (ESPEN) guidelines currently do not support the use of glutamine, arginine, and omega-3 fatty acids in well-nourished patients; however, supplementation may be indicated in malnourished patients or patients that are unable to be fed enterally [57].

**Summary and recommendation**: Due to the lack of evidence, the use of immunonutrition in hepatic surgery is not recommended yet.

**Evidence level**: Low.

**Grade of recommendation**: Weak.

### Preoperative fasting and preoperative carbohydrate load

The ESPEN and the American Society of Anesthesiologists guidelines currently recommend fasting for solids for 6 h before anesthesia and for liquids no more than 2 h before anesthesia [57, 72].

The purpose of giving carbohydrate drinks the evening before and 2–4 h before surgery is to ensure hydration and to reduce insulin resistance [73, 74]. Preoperative carbohydrate drinks have been associated with reduced anxiety, postoperative nausea and vomiting, postoperative insulin resistance, and length of hospitalization [57]. Preoperative carbohydrate loading reduces the resistance to insulin after liver surgery [75–77]. Moreover, Kobayashi et al. [78] found that giving late-evening carbohydrate load and an amino acid snack preoperatively improved the nutritional status of patients with perturbation of liver function. A Cochrane meta-analysis found that carbohydrate loading before elective surgery (18 studies for abdominal surgery) allowed a small reduction of LoS [79]. No difference in terms of complications was found. In a network meta-analysis published in 2017, carbohydrate loading before surgery was associated with a reduction of LoS in patients who were fasting, but did not show any benefit compared to water or placebo [80]. A 2010 RCT on major abdominal surgery (including liver surgery) found that preoperative carbohydrate loading did not improve postoperative outcomes [81]. However, patients with carbohydrate loading who underwent open surgery without epidural analgesia had a trend toward a shorter median LoS (7 vs. 9 days, *p* = 0.054) [81]. A recent systematic review of RCT in general surgery concluded that carbohydrate loading up to 2 h prior to surgery was safe and could decrease insulin resistance [82]. Some data support the deleterious effect of insulin resistance on liver regeneration [83]. Type 1 diabetes or active gastroesophageal reflux is relative contraindication to carbohydrate loading in the 2–4 h period before surgery although type 2 diabetes can receive it [84].

**Summary and recommendation:** Preoperative fasting of 2 h for liquids and 6 h for solids before anesthesia is safe and can be recommended. Carbohydrate loading is recommended the evening before liver surgery and 2–4 h before induction of anesthesia. Preoperative carbohydrate loading is safe and improves perioperative insulin resistance, but it is not clear if it is associated with a reduction of length of stay in liver surgery.

**Evidence level:** Preoperative fasting: moderate, carbohydrate loading: low.

**Grade of recommendation:** Preoperative fasting: strong, carbohydrate loading: weak.

### Pre-anesthetic medication

Pre-anesthetic medication has traditionally been given to allay anxiety, but long-acting agents impair psychomotor recovery after general anesthesia. A Cochrane review on anxiolytic premedication for outpatient surgery showed that patients who received oral anxiolytics had psychomotor function impairment 4 h after surgery, reducing their ability to ambulate, eat, and drink [85]. In selected cases, short-acting anxiolytics (such as 1–2 mg midazolam) can be given to ease regional anesthesia before general anesthesia induction. The American Geriatrics Society Beers Criteria for potentially inappropriate medication use in older patient populations (aged 65 years and older) strongly advise against using benzodiazepines as they may cause cognitive impairment and increase the risk of delirium and falls in the elderly [86].

More recently, preoperative medication was more commonly used as perioperative multimodal analgesic adjuncts. In liver surgery, the use of nonsteroidal anti-inflammatory drugs (NSAIDS) preoperatively is not recommended because of the risk of acute kidney injury. A meta-analysis of 281 trials (*n* = 24,682 participants) examining the use of gabapentinoids in major surgery showed that although there was a mild analgesic effect, there were significant problems with blurred vision and dizziness [87]. These appeared to be dose-related but could occur with normal dosing in the elderly, so their use in liver surgery was not recommended. Acetaminophen has to be dose-adjusted if significant liver parenchyma is removed. Scopolamine patches are effective in patients with high risk of postoperative nausea and vomiting (PONV) but should be avoided in the elderly due to central effects [88].

**Summary and recommendation:** Long-acting anxiolytic drugs should be avoided, particularly in the elderly. Preoperative gabapentinoids and nonsteroidal anti-inflammatory drugs are not recommended. Preoperative acetaminophen should be dose-adjusted according to extent of resection. Preoperative hyoscine patches can be used in patients with high risk for postoperative nausea and vomiting but should be avoided in the elderly.

**Evidence level:** Moderate.

**Grade of recommendation:** Strong.

### Anti-thrombotic prophylaxis

Liver surgery is an independent risk factor for postoperative thromboembolic events, and this risk is directly proportional to the magnitude of hepatectomy [89]. In addition, this risk extends beyond hospital discharge. Since the last published ERAS liver guidelines, one prospective study on the use of enoxaparin treatment in patients after curative HPB surgery for malignancies was published [90]. In this prospective multicenter study including 74 hepatectomies and 35 pancreaticoduodenectomies, subcutaneous injection of enoxaparin was initiated 48–72 h after surgery and repeated for 8 days. Neither major bleeding (primary endpoint) nor symptomatic venous thromboembolism (VTE) was observed. In a recent meta-analysis of 5 retrospective studies including 2256 patients who received chemical thromboprophylaxis, 1412 with mechanical prophylaxis only, the use of chemical thromboprophylaxis reduced the VTE incidence (2.6% vs. 4.6%) following liver surgery without any apparent risk of bleeding [91]. However, chemical thromboprophylaxis dosing was varied and was initiated at different times of the perioperative pathway. The results of the updated Cochrane review (7 RCT, 1,728 participants) on the use of prolonged thromboprophylaxis with low molecular weight heparin (LMWH) in abdominal and pelvic surgery favor the use of prolonged LMWH ≥14 days after surgery [92]. The overall incidence of VTE was reduced from 13.2% in the control group (i.e., only hospital thromboprophylaxis) to 5.3% in the study group. This reduction was also observed when analyzing the rate of deep venous thrombosis and symptomatic VTE. There is no clear evidence to start chemical thromboprophylaxis the day before liver surgery, although this issue has never been addressed in liver surgery studies. There are no reports suggesting that omission of the preoperative dose places patients at higher risk for VTE after hepatectomy. Although no specific studies for liver surgery were found, the use of intermittent pneumatic compression devices applied prior to induction of anesthesia combined with chemical thromboprophylaxis is supported in the literature [93–95]. A meta-analysis of 16,164 patients from 70 studies found almost a 50% risk reduction of VTE when combining intermittent pneumatic compression and chemical thromboprophylaxis compared to intermittent pneumatic compression alone (RR 0.54, 95% CI 0.32–0.91, *p* = 0.02) [93].

**Summary and recommendation:** Low molecular weight heparin or unfragmented heparin reduces the risk of thromboembolic events and should be routinely started postoperatively unless exceptional circumstances make this unsafe. Intermittent pneumatic compression devices should be used to further reduce this risk.

**Evidence level:** Use of low molecular weight heparin or unfragmented heparin: moderate, use of intermittent pneumatic compression devices: moderate.

**Grade of recommendation:** Use of low molecular weight heparin or unfragmented heparin: strong, use of intermittent pneumatic compression devices: strong.

### Preoperative steroids administration

According to a recently published meta-analysis including 6 RCT (*n* = 411 patients) focusing on liver surgery, the preoperative administration of steroids compared to placebo was not associated with a significant difference in the incidence of postoperative complications or LoS [96]. Of note, most of the studies included in the previous meta-analysis had relatively small sample sizes (*n* = 20–200, range of patients included) and lacked long-term follow-up data [96]. A supplementary double-blind RCT on 124 patients with laparoscopic liver surgery, which was not included in the previous meta-analysis, found similar results [97]. The doses used ranged from 500 to 30 mg/kg [97]. Both the above-cited studies reported that short-term administration of steroids before the operation was associated with reduction of surgical stress following liver resection, measured by IL-6 and C-reactive protein (both significantly reduced on postoperative day 1 in the steroids group compared to control) [96, 97]. Moreover, a recent RCT (*n* = 151 patients) comparing the preoperative use of 500 mg of methylprednisolone versus placebo showed that the methylprednisolone group had fewer postoperative complications (31% vs. 47%, *p* = 0.042), and in particular a lower rate of organ space SSI (7% vs. 18%, *p* = 0.036) [98].

Although preoperative administration of steroids in liver resection may promote the recovery of liver function, its systematic use in diabetic patients remains unresolved.

**Summary and recommendation:** Steroid administration (methylprednisolone at a dose of 500 mg) is recommended. No recommendation can be formulated on diabetic patients undergoing liver surgery.

**Evidence level:** Moderate.

**Grade of recommendation:** Weak.

### Antimicrobial prophylaxis and skin preparation

SSI and wound complications after liver surgery are associated with increased mortality, morbidity, hospital stay, and costs [99–102]*.* Despite recommendations to administer antibiotics in liver surgery before skin incision, hard data are lacking [103]. The highest level of evidence is offered by a recent network meta-analysis on 5 RCT accumulating 701 patients, from 1998 to 2016, comparing 4 antibiotic prophylaxis strategies (preoperative or postoperative short-duration, postoperative long-duration or no antibiotic prophylaxis) and their combination [104]. Surprisingly, the lowest rate of SSI was demonstrated for patients who received no antibiotic prophylaxis. However, this observation should be mitigated by the fact that the “no antibiotic prophylaxis” strategy was reported in only one of the RCT, a single-center study with moderate risk of bias, enrolling 120 patients for open hepatectomy without bile duct resection [105].

If a prophylactic antibiotic regimen is delivered, its duration should not exceed 24 h: This statement is based on the results of 2 Japanese trials with 670 patients undergoing open liver surgery without biliary reconstruction [106, 107]. The authors observed no difference in the incidence of SSI with a 1-day versus 3-day antibiotic regimen.

In case of complex surgery requiring biliary reconstruction, the frequency of SSI seems to be decreased with a targeted regimen (based on preoperative bile culture) compared to standard antibiotic treatment [108]. In this particular case, and based on the findings from an open-label single-center RCT of 86 patients, 2-day administration of antimicrobial prophylaxis offers similar results to a 4-day regimen, with respect to the absence of infectious complications or sepsis [109]. In this context, it has not been evaluated if preoperative or 1-day antibioprophylaxis is equivalent to 2-day antimicrobial prophylaxis. Moreover, preoperative biliary stenting might also be associated with SSI occurrence, but no robust data exist on the role of antibiotic prophylaxis in these patients [110, 111].

Regarding skin preparation, 2 robust trials comparing 2 different strategies were published during the last decade [112, 113]. A double-blind, single-center RCT including 100 patients undergoing liver surgery assessed the efficacy of a pre-disinfection process with chlorhexidine gluconate (CHG) *vs.* saline, before the application of povidone–alcohol solution (in both groups) [112]. No differences were observed in terms of postoperative SSI. One supplementary double-blind trial compared the efficacy of CHG–alcohol solution versus povidone-iodine for the prevention of SSI, on a heterogeneous target population (upper and lower gastrointestinal, biliary, thoracic and urogynecology or hepatobiliary and gastroesophageal surgeries) [113]. This large (*n* = 897), multicenter, international, and high-quality trial [113] reported a decrease in the rates of SSI infection in the CHG–alcohol solution group.

**Summary and recommendation:** Antibiotic prophylaxis (such as cefazolin) within 60 min before surgical incision is recommended, with no benefit extending it into the postoperative period. In case of complex liver surgery with biliary reconstruction, a targeted antibiotic pre-emptive regimen based on preoperative bile culture may be recommended, but its duration is unknown. Skin preparation with chlorhexidine-alcoholic solution is associated with a lower rate of surgical site infections, compared to povidone-iodine solution.

**Evidence level:** Antibiotics: moderate, skin preparation: moderate.

**Grade of recommendation:** Antibiotics: weak, skin preparation: strong.

### Minimally invasive approach

Two international consensus conferences (Louisville and Morioka) [114, 115] followed by one European consensus guideline (Southampton) [116] emphasized the benefits of laparoscopic liver resection for both benign and malignant tumors, including primary and metastatic diseases.

Regarding minor liver resections, 2 RCT focusing on colorectal liver metastases have demonstrated the short-term benefits of laparoscopy compared to open surgery, especially leading to a lower rate of morbidity, shorter hospital stay, lower postoperative morphine consumption, and better quality of life [117, 118].

Major hepatectomies performed by laparoscopy have not been assessed by RCT. However, several meta-analyses and propensity score studies showed potential short-term advantages in trained teams [119–122]. Even complex procedures such as staged hepatectomies for liver metastases have been reported and showed not only short-term advantage but also shorter delay between the postoperative course and chemotherapy restart [123]. Recently, a European experience from 9 tertiary referral centers reported lower bleeding, shorter hospital stay, and lower postoperative morbidity for both left and right hemihepatectomies [124]. These results need to be confirmed in future RCT.

Finally, in the setting of a living donor, the laparoscopic approach has not been evaluated in a controlled trial given the small number of performed procedures that would lead to insufficient patient recruitment. However, international registries and propensity score studies demonstrated postoperative advantages of laparoscopy, especially for left sectionectomy. For this indication, the risk of laparoscopic approach has been shown to be lower than donor nephrectomy. In expert centers, consensus conferences have claimed the benefit of laparoscopy for pediatric living liver donor transplantation, and a recent international registry showed the benefit of laparoscopy in major hepatectomies [115, 125–127].

Currently, data on robotic minimally invasive liver resection from RCT are lacking.

**Summary and recommendation**: In trained teams and when clinically appropriate, laparoscopic liver resection is recommended since it reduces postoperative length of stay and complication rates.

**Evidence level**: Moderate.

**Grade of recommendation**: Strong.

### Epidural, postoperative intravenous, and postoperative per oral analgesia

The advantage of thoracic epidural analgesia (TEA) is that it can modify the stress response as measured by biomarkers, which may have the potential to improve downstream oncological outcomes as shown in a RCT of 62 patients [128]. The sympathectomy from TEA can induce hypotension due to vasodilation, which can complicate fluid therapy, necessitate the need for low-dose vasopressors, and compound the risk of acute kidney injury [129]. In liver surgery, the postoperative prolongation of prothrombin time can make timing of removal of the epidural catheter problematic [130]. A RCT of 140 patients comparing TEA to intravenous patient-controlled analgesia (PCA) in hepatopancreatobiliary surgery found that TEA had better pain control and less use of opioids with similar LoS and complications in both groups [131]. A Cochrane analysis of 32 studies (1716 patients) of TEA *vs.* PCA opiate in open surgery showed a slightly better pain reduction with TEA but increased risk of technical failure, more frequent episodes of hypotension, and more pruritus [132]. Evidence level was graded as moderate.

Intrathecal opiates have been used to reduce opiate requirement postoperatively when combined with a multimodal analgesic regimen and avoid the need for continuous infusions [133, 134]. A recent review of 11 studies confirmed this technique to have similar results to TEA but with a lower likelihood of postoperative hypotension and a reduced LoS [134]. In a 2017 RCT (56 patients), addition of a selective COX-2 inhibitor (parecoxib, unauthorized in certain countries) to PCA for patients undergoing open liver resection was found to decrease postoperative pain compared to PCA alone [135]. For open living donor hepatectomy, the use of ketorolac infusion in addition to intravenous fentanyl PCA improved postoperative analgesia and decreased the dose of used fentanyl as shown in a RCT of 60 patients [136]. Therapeutic acetaminophen has been shown to be safe after major hepatectomy if liver function was preserved [137]. However, it is prudent to reduce the dose to 2 g per day if significant liver parenchyma is resected. Postoperatively, NSAIDS should be used only if renal function is normal.

The analgesic requirements after laparoscopic surgery combined with earlier gut function enable analgesia to be achieved by oral route soon after surgery. This and the smaller incisions (the main one being to deliver the specimen) reduced the need for regional analgesic techniques. In laparoscopic liver surgery, a RCT of 124 patients showed that an intravenous infusion pump of parecoxib provided superior analgesia and fewer adverse outcomes compared to an intravenous infusion pump of fentanyl [138].

**Summary and recommendation:** For open liver surgery, thoracic epidural analgesia can provide excellent analgesia but has significant disadvantages. In fact, optimal postoperative management is key to avoid hypotension and mobility issues which can be detrimental to rapid recovery. Multimodal analgesia (including potential use of intrathecal opiates) is recommended. Regarding laparoscopic surgery, there is no need for regional anesthesia techniques, as multimodal analgesia combined with judicious intravenous opiates provide functional analgesia.

**Evidence level:** Open (multimodal analgesia): high, laparoscopy (multimodal analgesia): low.

**Grade of recommendation:** Open (multimodal analgesia): strong, laparoscopy (multimodal analgesia): weak.

### Wound catheter and transversus abdominis plane (TAP) block

Continuous wound infiltration (CWI) of local anesthetic using wound catheters has been compared with TEA in a number of RCT in patients undergoing liver surgery. The Liver 1 trial (RCT, *n* = 65) compared CWI to TEA and showed similar static pain scores, better dynamic and early pain scores with TEA and shorter LoS with CWI [139]. No difference was found in terms of mobilization and overall complication rate. A similar RCT of 83 patients showed equivalent pain scores and reduced treatment failure for CWI but did not show reduced LoS [140]. The Liver 2 trial (RCT, *n* = 93) combined CWI with TAP blocks and compared it with TEA [141]. This trial showed equivalent static and dynamic pain scores in both arms both early and later after surgery and earlier discharge from hospital in the CWI and TAP block group. A further multicentric RCT of 105 patients containing patients undergoing liver resection and other HPB procedures showed non-inferiority in analgesia between CWI and TEA [142]. Other RCT (sample size range: 40–153) using ropivacaine as a continuous infusion in CWI showed reduction in opiate requirements and/or reduced LoS in patients receiving CWI [143–149]. One RCT of 99 patients allocated patients after liver resection who received patient-controlled opiate analgesia to either ropivacaine CWI infusion or placebo and showed no difference in pain scores or LoS between the groups [150]. Two RCT have compared TAP blocks to TEA in abdominal surgery; the first (*n* = 62) showed equivalent pain scores but higher opiate requirements in the TAP group [151]. The second RCT (*n* = 65) in major general cancer resections showed lower opiate requirements in the TAP group and less frequent postoperative hypotension [152]. A further 3 RCT of TAP blocks showed reduced opiate requirements in patients undergoing liver surgery in different contexts [153–155]. Two RCT in patients with cirrhosis undergoing liver surgery also showed benefit of TAP blocks in reducing opiate requirements of patients [156, 157]. A recent RCT of 63 patients showed benefit from regional blockade using quadratus lumborum blocks in patients undergoing liver resection [158]. There is one RCT reported which looked at the use of sponges placed over port sites from minimally invasive liver surgery soaked with ropivacaine *vs.* placebo which showed benefit in pain scores from the ropivacaine group [159].

Pooled data in 2 meta-analyses of patients undergoing major abdominal open surgery (inclusion of 16 RCT) [160] and specifically liver resections (inclusion of 3 RCT) [161] compared outcomes of patients receiving CWI *vs*. TEA, and both showed lower complication rates and faster recovery after surgery with CWI but slightly better pain control with TEA at certain time points following surgery.

**Summary and recommendation:** Continuous local anesthetic wound infiltration provides lower complication rates and overall equivalent analgesia to thoracic epidural analgesia. Local anesthetic transversus abdominis plane blockade as a supplement to standard analgesia improves pain control and reduces opiate usage.

**Evidence level:** High.

**Grade of recommendation:** Strong.

### Prophylactic nasogastric intubation

Historically, nasogastric tubes (NGTs) were routinely placed after major abdominal surgery due to concerns for postoperative abdominal distention, nausea, and vomiting. The benefits of this approach, however, were questionable. Two recent studies focusing on hepatectomy patients have added to the body of literature that speaks against routine prophylactic nasogastric intubation [162, 163]. The first is a RCT that showed no difference in the rates of overall morbidity, pulmonary complications, postoperative vomiting, time to oral intake, and hospital LoS between patients randomized to a NGT group *vs*. a no-NGT group [162]. The second is a systematic review and meta-analysis with over 1300 hepatectomy patients from 7 RCT and likewise demonstrated no benefits to NGT with regard to return of bowel function [163]. In fact, NGT was associated with greater LoS and delay to starting diet. Taken together in the context of the existing literature, these studies suggest that routine prophylactic NGT should be discouraged.

**Summary and recommendation:** Prophylactic nasogastric intubation does not offer postoperative benefits and may in fact increase hospital length of stay. Routine use of prophylactic nasogastric intubation is not recommended.

**Evidence level:** High.

**Grade of recommendation:** Strong.

### Prophylactic abdominal drainage

The existing literature on the topic of prophylactic abdominal drainage after liver surgery has been inconclusive at best to this point, with contradictory results. More recently, several studies have emerged that speak against this practice [164–168]. A RCT found no improvement in postoperative morbidity with abdominal drainage after hepatectomy; instead, the drain was associated with some inherent complications such as bleeding and infection, especially among patients with chronic liver diseases [164]. Likewise, an analysis of 199 patients undergoing liver surgery found that intraoperative placement of perihepatic drainage failed to decrease rates of perioperative complications [165]. In addition, it should be noted that selection bias likely tainted the outcomes of this study, as drains were more likely to be placed for more complex tumors and resections. More recently, a review by Messager et al. [166] on the topic of prophylactic intra-abdominal drainage in elective major gastrointestinal surgery concluded that, based on high-quality evidence, no argument could be made for routine abdominal drainage following hepatic resection without biliary anastomosis. Finally, a systematic review and meta-analysis reviewed results from 6 RCT including 665 patients; its main finding was that routine use of abdominal drains after elective uncomplicated liver surgery was not associated with a reduction in postoperative complications [167]. Rather than routine abdominal drainage, clinicians may wish to consider other maneuvers to reduce the rate of post-hepatectomy complications such as bile leaks and organ space infections. One such practice is the systematic use of an intraoperative air leak test after major hepatectomy [169, 170].

**Summary and recommendation:** The routine use of abdominal drain placement is not indicated for hepatectomy without biliary reconstruction. No recommendation can be made for hepatectomy with biliary reconstruction.

**Evidence level:** High.

**Grade of recommendation:** Strong.

### Preventing intraoperative hypothermia

Maintaining body temperature >36 °C has been recommended to reduce both cardiac and non-cardiac complications [171–176]. One meta-analysis [172] of 67 trials has shown that mild hypothermia was associated with increased SSI and blood loss. There is a lack of studies relating specifically to liver surgery. One meta-analysis of 23 trials comparing warming systems suggested that circulating water-based systems offer better warming than forced air systems [177] although a recent retrospective study (*n* = 50) has used a multimodal approach to temperature management during surgery with good temperature control [178]. A systematic review of 22 studies has suggested heating insufflated gases in laparoscopic abdominal surgery to maintain body temperature but patient outcomes were not improved [179]. Liver cooling techniques have been used to minimize ischemia reperfusion injury to the liver, but temperature changes in the whole body in such procedures have not been reported [180].

**Summary and recommendation:** Perioperative normothermia using multimodal temperature management (including circulating water garments or forced warm air) should be maintained during open and minimally invasive liver surgery.

**Evidence level:** Moderate.

**Grade of recommendation:** Strong.

### Postoperative artificial nutrition and early oral intake

Early oral diet has been shown to be safe and to decrease the time to first bowel movement in abdominal and liver surgery [181]. The ESPEN guidelines on clinical nutrition in surgery published in 2017 recommend oral nutrition postoperatively [57]. Moreover, full nutritional assessment preoperatively is required before liver surgery to identify malnutrition and to better tailor the nutritional management [182, 183]. Nutrient deficiency should be corrected before, during, and after liver surgery according to identified deficits.

Oral nutrition was found to be better than total parenteral nutrition (TPN) after hepatectomy in a study of 32 patients [184]. Ishikawa et al. [185] randomly assigned 24 patients to usual oral diet *vs.* oral and parenteral diet (preoperatively and during 7 postoperative days). No difference in terms of complications was found.

A review based on 6 articles recently showed that artificial (i.e., enteral and parenteral) nutrition in liver surgery is poorly defined and that it should not be routinely given [186]. Patients with malnutrition or complications inducing fasting or appetite loss might benefit from artificial nutrition. If artificial nutrition support is considered, Gao et al. [187] in a meta-analysis (9 studies) showed that enteral nutrition (EN) induced better outcomes than TPN. In a systematic review and meta-analysis including 18 RCT with 2540 patients, Zhao et al. [188] assessed TPN *vs*. EN in major abdominal surgery for cancer. The authors found better outcomes with EN (decreased LoS and first flatus time, increased serum albumin level).

A more general review on patients with liver disease by Sun et al. [189] recommended that if a patient has a need for nutritional support, EN should first be considered. EN can then be complemented with parenteral nutrition when EN cannot bring all energy needs (60%). Nutritional support should be individualized based on patient need, disease characteristics, liver tolerance, and integrity of the gastrointestinal tract.

**Summary and recommendation:** Early oral intake with normal diet should be implemented after hepatectomy. Individualized need for artificial nutrition should be assessed for malnourished patients, patients with complications causing several days of fasting, and patients with liver cirrhosis. If artificial nutrition is considered, enteral administration should be preferred.

**Evidence level:** High.

**Grade of recommendation:** Strong.

### Postoperative glycemic control

Perioperative hyperglycemia is frequently observed after major surgery [190, 191]. The cause of perioperative hyperglycemia is a transitory resistance to insulin leading to a decreased peripheral uptake of glucose [192]. Surgical stress produces a raise in blood sugar, which modifies hepatic metabolism regulation and immune function, impacting the recovery after surgery. In patients undergoing colorectal and pancreatic surgery, hyperglycemia occurring in the initial postoperative days is related to postoperative complications [193, 194]. Sensitivity to insulin after surgery is significantly decreased when insulin is not used intraoperatively to treat hyperglycemia [195]. Moreover, the glucose concentration rapidly changes during liver resection using the Pringle maneuver, because hypoxia induces glycogen breakdown within hepatocytes [196]. One RCT including 88 patients undergoing hepatectomy compared the use of an artificial pancreas (closed-loop glycemic control system) to the usual sliding-scale method for insulin therapy and showed that SSI and total hospital costs were decreased in patients who were treated with closed-loop glycemic control system [197]. It has also been shown that supplementation with carbohydrates and branched-chain amino acid-enriched nutrients before liver surgery lowers the resistance to insulin [77]. Additionally, elevated serum lactate after hepatectomy that has been shown to be associated with postoperative complications is linked to insulin resistance and/or ischemia–reperfusion injury [198]. In some RCT, high-dose insulin therapy or intensive insulin therapy reduced postoperative liver dysfunction, infections, and complications and improved the liver glycogen content in patients undergoing HPB surgery [199–201]. One RCT showed that perioperative glucose and insulin administration more effectively resulted in normoglycemia than did the standard insulin therapy for patients undergoing liver resection [202]. A systematic review of intensive insulin therapy showed that high blood sugar was not predictive of SSI among diabetic patients and recommended a target blood glucose level of <150 mg/dl (<8.3 mmol/l) in patients without diabetes undergoing gastroenterological surgery [203]. A conventional protocol is indicated for patients admitted to the general ward (not the intensive care unit) to avoid the risk of hypoglycemia [203]. According to one recent RCT, preoperative administration of liraglutide stabilized the perioperative plasma glucose level and reduced the perioperative insulin requirement without increasing the risk of hypoglycemia [204].

**Summary and recommendation:** Insulin therapy for maintenance of normoglycemia (<8.3 mmol/l) is recommended.

**Evidence level:** High.

**Grade of recommendation:** Strong.

### Prevention of delayed gastric emptying (DGE)

Left hepatic resection might induce a higher DGE risk (15–20%) because of disturbance of regular gastrointestinal motion at the contact plane between the stomach and the surface of the cut liver [205, 206]. One RCT (*n* = 40) found no difference of DGE incidence if an omental flap to cover the liver cut surface after left-sided liver resection was used [205], whereas another RCT (*n* = 49) found a reduced incidence of DGE when using an omental flap [206]. In addition, one cross-sectional study (*n* = 42, 15 patients in the fixation group) showed that fixation of the round ligament reduced the incidence of DGE [207].

**Summary and recommendation:** Use of an omental flap to cover the cut surface of the liver might reduce the risk of delayed gastric emptying after left-sided liver resection.

**Evidence level:** Low.

**Grade of recommendation:** Weak.

### Stimulation of bowel movement

Shimada et al. [208] found in a multicenter RCT that daikenchuto (TU-100, traditional herbal medicine) significantly decreased the median time to first bowel movement by 5 h in 231 patients who underwent hepatectomy for cancer. Although significant, this 5-h difference is probably not clinically relevant as complication rates were similar in both groups. Another RCT assessing the effect of daikenchuto after hepatectomy found that the daikenchuto group had shorter time to bowel movement and oral intake, but complications were similar [209]. You et al. [210] performed a 3-arm RCT to assess ileus rates in patients with HCC undergoing liver resection. Simo decoction (traditional Chinese herbal medicine) with acupuncture was compared to gum chewing and no specific postoperative intervention (control group). Both interventions were found to diminish the time to first stool compared to the control group, whereas only the group with simo decoction and acupuncture had a shorter length of hospital stay. In a RCT of 68 patients undergoing liver surgery, the group with laxatives had reduced time to passage of stool but similar secondary outcomes, such as DGE, LoS, or time to functional recovery [211]. Jang et al. [212] showed in a prospective case–control study including 42 patients that gum chewing permitted to decrease the time to first flatus and the xerostomia rate, but did not have an effect on LoS or analgesic use.

**Summary and recommendation:** Postoperative laxatives, gum chewing, herbal medicine, or decoction after hepatectomy might reduce the time to first flatus or stool but do not impact the morbidity rate. Current data do not permit the recommendation of the routine use of postoperative laxatives, gum chewing, herbal medicine, or decoction to stimulate bowel movement after liver surgery.

**Evidence level:** Moderate.

**Grade of recommendation:** Weak.

### Early and scheduled mobilization

Bed rest is associated with multiple established deleterious effects including muscle atrophy, thromboembolic disease, and insulin resistance [213–215]. A RCT involving 120 patients undergoing liver resection showed a significantly faster postoperative gastrointestinal function and shorter length of hospital stay after performing early activity (from postoperative day 1) [216]. An early postoperative mobilization program based on supervised exercises improved functional capacity in patients undergoing major elective abdominal oncologic surgery [217].

So far, however, no consensus has been defined regarding the type, frequency, and intensity of physical therapy in liver surgery [218].

**Summary and recommendation:** Early mobilization (out of bed) after liver surgery should be established from the operative day until hospital discharge. No recommendation can be made regarding the optimal duration of mobilization.

**Evidence level:** Moderate.

**Grade of recommendation:** Strong.

### Postoperative nausea and vomiting (PONV) prophylaxis

PONV occurs frequently after major surgery (25–30%). The multimodal approach and opioid reduction provided by ERAS enable the majority of patients to eat early after hepatectomy [219]. Known risk factors, such as previous PONV, female gender, younger age, non-smoker, and use of volatile anesthetic agents and opioids, should be evaluated before the operation [220]. The 5-HT_3_ antagonists are the primary treatment because of their safe side effect profile. Low-dose dexamethasone is a good additive preventative agent and facilitates hepatic regeneration [221]. Of note, there is no supplementary advantage of using higher doses [221]. However, dexamethasone should be used with caution in diabetics as it can transiently worsen glycemic control [222]. Antihistamines, butyrophenones, and phenothiazines can also be used as second-line therapy [88]. The international consensus group on PONV recommends using 2 antiemetic drugs to decrease PONV and to improve efficacy [88]. Table 3 summarizes potential antiemetic drugs with doses and timing of use.

**Table 3:** Antiemetic drugs for postoperative nausea and vomiting (PONV) prophylaxis with doses and timing of use

Medication	Doses	Timing of use
Ondansetron	4 mg IV	End of surgery
Dexamethasone	4–8 mg IV	At induction
Droperidol	0.625 mg IV	End of surgery
Metoclopramide	10 mg IV/PO	Postoperatively
Scopolamine	1 mg transdermal patch	Before surgery

This table is based on the recommendations from the international consensus group on PONV

IV: intravenous, PO: per os

**Summary and recommendation:** A multimodal approach to postoperative nausea and vomiting should be used. Patients should receive postoperative nausea and vomiting prophylaxis with at least 2 antiemetic drugs such as dexamethasone and ondansetron.

**Evidence level:** High.

**Grade of recommendation:** Strong.

### Fluid management

Blood loss and transfusion rates remain central risk factors leading to higher morbidity and mortality after liver resections [223–225]. A Cochrane review showed that a lower central venous pressure (CVP) decreased blood loss, but without significant difference in red blood cell transfusion requirements, intraoperative morbidity, or long-term survival benefits [226]. Another systematic review and meta-analysis also confirmed that low CVP was associated with less blood loss [227].

Regarding fluid management for major hepatic surgery, there is currently no protocol available providing the optimum amount of fluid to be given to patients. The current concept must focus on the maintenance of central euvolemia, thereby preventing any excess of salt or water. Goal-directed fluid therapy (GDFT), targeting adequate cardiac output and end-organ perfusion, has attracted much attention. A meta-analysis of 32 RCT including about 3000 patients testing the impact of GDFT during major surgery, i.e., not only liver surgery, demonstrated significant benefits in reducing morbidity and mortality [228]. A RCT published in 2015 found that stroke volume variation (SVV)-guided GDFT compared to standard fluid resuscitation decreased the intraoperative infused fluid volume without decreasing postoperative complications [229]. On multivariable analysis, higher intraoperative fluid volume was an independent risk factor for 30-day morbidity. Recently, Weinberg et al. [230] showed in an RCT that a restrictive GDFT did not decrease LoS and fluid-related complications compared to conventional care within an ERAS pathway for major liver resection. Of note, only 24 patients were included in each arm.

Excessive administration of crystalloids should be avoided as much as blood loss during liver surgery. To guide fluid management during surgery, the measurement of SVV has been proposed to replace CVP monitoring [231]. A randomized prospective trial comparing SVV monitoring versus CVP recording in 90 patients undergoing laparoscopic liver surgery showed a reduced conversion rate as well as reduced blood loss in favor of the SVV approach [232]. The choice for intravenous fluid therapy in liver surgery is still under debate. A systematic review covering 43 RCT compared 18 fluid types (9 crystalloids and 9 colloids) in major abdominal surgery concluded that the best approach was balanced crystalloids (e.g., Ringer’s lactate) as maintenance fluid and colloids as volume expander (e.g., human albumin) [233]. Concerning the postoperative period, a recent retrospective study showed that a weight gain ≥3.5 kg on postoperative day 2 was an independent risk factor for major complication after liver surgery [234]. This suggests that postoperative weight fluctuation should be carefully monitored and potentially minimized.

**Summary and recommendation**: Low central venous pressure (below 5 cm H_2_O) with close monitoring is recommended during hepatic transection. As maintenance fluid balanced crystalloid should be preferred over 0.9% saline or colloids. Goal-directed fluid therapy optimizes cardiac output and end-organ perfusion. This may be particularly beneficial after the intraoperative liver resection during a low central venous pressure state to restore tissue perfusion. Patients who have comorbidities and reduced cardiac function may benefit most.

**Evidence level:** High.

**Grade of recommendation:** Strong.

### Monitoring/audit

Monitoring the outcomes after implementation of ERAS allows performing a precise audit. Outcome monitoring therefore represents the first step to establish an audit of quality. A recent study reported the successful implementation of a nationwide audit for liver surgery in the Netherlands [235]. This audit on postoperative outcomes after liver surgery was intended to evaluate the quality of centers performing these operations and to reach or maintain the best surgical quality. Otherwise, no study specifically designed for liver surgery has been published yet. A Cochrane systematic review on the effects of audit and feedback analyzed 140 studies [236]. It was found that audit and feedback generally induce improvements. The audit was more efficient when the baseline performance was low. The structure of the audit or feedback played a role. It was, for example, of interest to identify specific targets and put in place a plan of action. Another review by Ivers et al. [237] revealed that the body of evidence showing that audit improves outcomes was substantial, but that progress and evolution in this field were not present in recent literature. To a larger scale such as healthcare system, Grimshaw et al. [238] recommended the implementation of laboratories to better understand the science behind audit and feedback and to improve audit and feedback and their impact. One article highlighted the importance of undertaking actions over just measurement [239]. Recently, the Clinical Performance Feedback Intervention Theory issued from a systematic review and meta-synthesis postulated that an effective feedback was a cyclical process and that every missing links stopping the “cycle” cause effect loss of the feedback [240]. This theory includes recommendations for optimally designing or implementing an audit intervention. Practical suggestions on how to effectively display or deliver feedback have also been published [241].

**Summary and recommendation:** Substantial literature exists supporting that audit and feedback improve outcomes in health care and surgery. Regular audit and feedback should be implemented and performed in liver surgery to monitor and improve postoperative outcomes and compliance to the ERAS program.

**Evidence level:** Moderate.

**Grade of recommendation:** Strong.

## Discussion

This systematic review and modified Delphi consensus elaborated 25 recommendations based on the best available evidence published until mid-2020. Nine items had a high level of evidence: preoperative smoking and alcohol cessation, preoperative nutrition, wound catheter and TAP block, prophylactic nasogastric intubation, prophylactic abdominal drainage, postoperative artificial nutrition and early oral intake, postoperative glycemic control, PONV prophylaxis, and fluid management.

Regarding differences with 2016 recommendations, more evidence regarding use of steroids before hepatectomy has been published since. It is now routinely recommended in non-diabetic patients. Routine drainage after hepatectomy without biliary reconstruction is not recommended in the present guidelines, whereas in 2016 no conclusive evidence and no recommendation for or against the use of drain were given. In addition, 3 novel items were introduced: prehabilitation, preoperative biliary drainage, and preoperative smoking and alcohol cessation. The novelties of these guidelines are the addition of the 3 novel items mentioned hereabove and the reassessment of the previously published items based on the most recent literature data.

It is not clear if patients with cirrhosis undergoing liver surgery should be managed differently within an ERAS program. Preliminary data showed that ERAS was safe in these patients [242, 243]. Further robust data are needed, and it remains unclear if ERAS pathways should be adapted in cirrhotic patients undergoing liver surgery.

It is important to mention that the compliance (adherence) to all ERAS items is paramount. It has been clearly shown that higher compliance to the ERAS pathway allows to have better postoperative outcomes compared to lower compliance [244, 245].

In conclusion, these guidelines for perioperative care after liver surgery were developed based on the best available evidence and recommend management for 25 perioperative items.

## Supplementary Information

Below is the link to the electronic supplementary material.

Supplementary file1 (DOCX 43 kb)
