# Supplementary material for: Guidelines for Perioperative Care for Liver Surgery: Enhanced Recovery After Surgery (ERAS) Society Recommendations 2022
Source: World J Surg. 2022 Oct 30;47(1):11–34. doi: 10.1007/s00268-022-06732-5 (PMC9726826; doi:10.1007/s00268-022-06732-5)
Supplement: Supplementary file 1 — Supplementary file1 (DOCX 43 kb) [file 268_2022_6732_MOESM1_ESM.docx]

Literature search strategies

| 1. Enhanced recovery after surgery (general search) | | | | |
| --- | --- | --- | --- | --- |
| Database | Search strategy | Date | References | |
|  |  |  | Found | After deduplication |
| Medline OVID SP | (Hepatectomy/ OR exp Liver/su OR exp Liver Diseases/su OR (Hepatectom* OR Hemihepatectom* OR ((Hepatic OR Liver) ADJ3 (Resection* OR Lobectom* OR surg* OR operation* OR segmentectom*))).ab,ti,kf.) AND ("Enhanced Recovery After Surgery"/ OR ((enhanced ADJ4 recovery) OR (ERAS ADJ3 (principles OR program* OR guideline* OR pathway*))).ab,ti,kf.) NOT (exp animals/ not humans.sh.)  limit to yr="2010 -Current" | 28.01.20 | 122 | 119 |
| Embase | ('liver surgery'/de OR 'liver resection'/exp OR 'liver disease'/exp/dm_su OR (Hepatectom* OR Hemihepatectom* OR ((Hepatic OR Liver) NEAR/3 (Resection* OR Lobectom* OR surg* OR operation* OR segmentectom*))):ab,ti,kw) AND ('enhanced recovery after surgery'/de OR ((enhanced NEXT/4 recovery) OR (ERAS NEAR/3 (principles OR program* OR guideline* OR pathway*))):ab,ti,kw) AND [english]/lim AND [2010-2020]/py NOT ([animals]/lim NOT [humans]/lim) NOT (‘conference abstract’/it OR ‘conference review’/it) | 28.01.20 | 140 | 36 |
| Cochrane Library | ((Hepatectom* OR Hemihepatectom* OR ((Hepatic OR Liver) NEAR/2 (Resection* OR Lobectom* OR surg* OR operation* OR segmentectom*))):ab,ti,kw) AND (((enhanced NEAR/3 recovery) OR (ERAS NEAR/2 (principles OR program* OR guideline* OR pathway*))):ab,ti,kw)  Filters : Conference abstracts : from 2018; Exclusion of clinical trials registry records | 28.01.20 | 39 | 7 |
| Total |  |  | 301 | 162 |

| 1. Preoperative counseling | | | | |
| --- | --- | --- | --- | --- |
| Database | Search strategy | Date | References | |
|  |  |  | Found | After deduplication |
| Medline OVID SP | (Hepatectomy/ OR ((exp Liver/su OR exp Liver Diseases/su) NOT Liver transplantation/) OR (Hepatectom* OR Hemihepatectom* OR ((Hepatic OR Liver) ADJ3 (Resection* OR Lobectom* OR surg* OR operation* OR segmentectom*))).ab,ti,kf.) AND (("Patient Education as Topic"/ AND ("Preoperative Period"/ OR "Preoperative Care"/)) OR (((preoperative* OR pre-operative OR before-surgery OR before-operation OR preadmission) ADJ5 (counselling OR counseling OR knowledge OR informati* OR visit OR education)) OR (patient ADJ5 (counselling OR counseling)) OR ((preoperative* OR pre-operative OR before-surgery OR before-operation OR preadmission) AND "patient education")).ab,ti,kf.) AND English.lg. not (exp animals/ not humans/) NOT (editorial/ or letter/)  limit to yr="2010 -Current" | 07.02.20 | 66 | 64 |
| Embase | ('liver surgery'/de OR 'liver resection'/exp OR ('liver disease'/exp/dm_su NOT 'liver transplantation'/exp) OR (Hepatectom* OR Hemihepatectom* OR ((Hepatic OR Liver) NEAR/3 (Resection* OR Lobectom* OR surg* OR operation* OR segmentectom*))):ab,ti,kw) AND ('preoperative education'/de OR 'patient counseling'/de OR ('patient education'/de AND 'preoperative period'/exp) OR (((preoperative* OR pre-operative OR before-surgery OR before-operation OR preadmission ) NEAR/5 (counselling OR counseling OR knowledge OR informati* OR visit OR education)) OR (patient NEAR/5 (counselling OR counseling)) OR ((preoperative* OR pre-operative OR before-surgery OR before-operation OR preadmission) AND "patient education")):ab,ti,kw) AND [english]/lim AND [2010-2020]/py NOT ([animals]/lim NOT [humans]/lim) NOT ('conference abstract'/it OR 'conference review'/it OR 'editorial'/it OR 'letter'/it) | 07.02.20 | 92 | 34 |
| Cochrane Library | (Hepatectom* OR Hemihepatectom* OR ((Hepatic OR Liver) NEAR/3 (Resection* OR Lobectom* OR surg* OR operation* OR segmentectom*))):ab,ti,kw AND (((preoperative* OR pre-operative OR before-surgery OR before-operation OR preadmission) NEAR/5 (counselling OR counseling OR knowledge OR informati* OR visit OR education)) OR (patient NEAR/5 (counselling OR counseling)) AND ((preoperative* OR pre-operative OR before-surgery OR before-operation OR preadmission) AND "patient education")):ab,ti,kw  Publication Year from 2010 to 2020 / NOT Conference abstract | 07.02.20 | 21 | 15 |
| Total |  | 07.02.20 | 179 | 113 |

| 1. Perioperative nutrition | | | | |
| --- | --- | --- | --- | --- |
| Database | Search strategy | Date | References | |
|  |  |  | Found | After deduplication |
| Medline OVID SP | (Hepatectomy/ OR ((exp Liver/su OR exp Liver Diseases/su) NOT Liver transplantation/) OR (Hepatectom* OR Hemihepatectom* OR ((Hepatic OR Liver) ADJ3 (Resection* OR Lobectom* OR surg* OR operation* OR segmentectom*))).ab,ti,kf.) AND (((exp Nutritional Support/ OR "Nutritional Status"/ OR Dietary Supplements/) AND (Preoperative Care/ OR Preoperative Period/ OR Perioperative Care/ OR exp Perioperative Period/ OR Perioperative Medicine/ OR exp Perioperative Care/ OR (preoperative* OR pre-operative OR perioperative OR peri-operative OR before-surgery OR before-operation).ab,ti,kf.)) OR (((nutrition* OR supplement*) ADJ5 (preoperative* OR pre-operative OR perioperative OR peri-operative)) OR malnourished OR malnutrition).ab,ti,kf.) AND English.lg. not (exp animals/ not humans/) NOT (editorial/ or letter/)  limit to yr="2010 -Current" | 07.02.20 | 112 | 112 |
| Embase | ('liver surgery'/de OR 'liver resection'/exp OR ('liver disease'/exp/dm_su NOT 'liver transplantation'/exp) OR (Hepatectom* OR Hemihepatectom* OR ((Hepatic OR Liver) NEAR/3 (Resection* OR Lobectom* OR surg* OR operation* OR segmentectom*))):ab,ti,kw) AND ((('nutritional support'/de OR 'nutrition'/exp OR 'dietary supplement'/de) AND ('preoperative period'/exp OR 'perioperative period'/de OR (preoperative* OR pre-operative OR perioperative OR peri-operative OR before-surgery OR before-operation):ab,ti,kw)) OR (((nutrition* OR supplement*) NEAR/5 (preoperative* OR pre-operative OR perioperative OR peri-operative)) OR malnourished OR malnutrition):ab,ti,kw) AND [english]/lim AND [2010-2020]/py NOT ([animals]/lim NOT [humans]/lim) NOT ('conference abstract'/it OR 'conference review'/it OR 'editorial'/it OR 'letter'/it) | 07.02.20 | 351 | 258 |
| Cochrane Library | (Hepatectom* OR Hemihepatectom* OR ((Hepatic OR Liver) NEAR/3 (Resection* OR Lobectom* OR surg* OR operation* OR segmentectom*))):ab,ti,kw AND (((nutrition* OR supplement*) NEAR/5 (preoperative* OR pre-operative OR perioperative OR peri-operative)) OR malnourished OR malnutrition):ab,ti,kw  Publication Year from 2010 to 2020 / NOT Conference abstract | 07.02.20 | 42 | 28 |
| Total |  | 07.02.20 | 505 | 398 |

| 1. Perioperative oral immunonutrition | | | | |
| --- | --- | --- | --- | --- |
| Database | Search strategy | Date | References | |
|  |  |  | Found | After deduplication |
| Medline OVID SP | (Hepatectomy/ OR ((exp Liver/su OR exp Liver Diseases/su) NOT Liver transplantation/) OR (Hepatectom* OR Hemihepatectom* OR ((Hepatic OR Liver) ADJ3 (Resection* OR Lobectom* OR surg* OR operation* OR segmentectom*))).ab,ti,kf.) AND (Exp "Arginine"/ OR exp "Fatty Acids, Omega-3"/ OR exp "Nucleotides"/ OR (Immunonutrition OR (immun* ADJ3 nutrition) OR imen OR arginine OR glutamine OR nucleotid OR "omega 3").ab,ti,kf.) AND English.lg. not (exp animals/ not humans/) NOT (editorial/ or letter/)  limit to yr="2010 -Current" | 07.02.20 | 130 | 130 |
| Embase | ('liver surgery'/de OR 'liver resection'/exp OR ('liver disease'/exp/dm_su NOT 'liver transplantation'/exp) OR (Hepatectom* OR Hemihepatectom* OR ((Hepatic OR Liver) NEAR/3 (Resection* OR Lobectom* OR surg* OR operation* OR segmentectom*))):ab,ti,kw) AND ('immunonutrition'/de OR 'arginine'/de OR 'glutamine'/de OR 'omega 3 fatty acid'/de OR 'nucleotide'/exp OR (Immunonutrition OR (immun* NEAR/3 nutrition) OR imen OR arginine OR glutamine OR nucleotid OR "omega 3"):ab,ti,kw) AND [english]/lim AND [2010-2020]/py NOT ([animals]/lim NOT [humans]/lim) NOT ('conference abstract'/it OR 'conference review'/it OR 'editorial'/it OR 'letter'/it) | 07.02.20 | 332 | 262 |
| Cochrane Library | (Hepatectom* OR Hemihepatectom* OR ((Hepatic OR Liver) NEAR/3 (Resection* OR Lobectom* OR surg* OR operation* OR segmentectom*))):ab,ti,kw and (Immunonutrition or (immun* near/3 nutrition) or imen or arginine or glutamine or nucleotid or "omega 3"):ab,ti,kw  Publication Year from 2010 to 2020 / NOT Conference abstract | 07.02.20 | 43 | 18 |
| Total |  | 07.02.20 | 505 | 410 |

| 1. Preoperative fasting and carbohydrates | | | | |
| --- | --- | --- | --- | --- |
| Database | Search strategy | Date | References | |
|  |  |  | Found | After deduplication |
| Medline OVID SP | (Hepatectomy/ OR ((exp Liver/su OR exp Liver Diseases/su) NOT Liver transplantation/) OR (Hepatectom* OR Hemihepatectom* OR ((Hepatic OR Liver) ADJ3 (Resection* OR Lobectom* OR surg* OR operation* OR segmentectom*))).ab,ti,kf.) AND (Fasting/ OR "Diet, Carbohydrate Loading"/ OR exp Carbohydrates/ad OR exp Dietary Carbohydrates/ad OR "Diet, Reducing"/ OR (fasting OR ((preoperative OR pre-operative OR overnight OR (before-surgery OR before-operation) ADJ3 fast*) OR ((low-calor* OR hypocaloric OR reducing) ADJ3 diet) OR ((preoperative OR pre-operative OR drink* OR beverage* OR loading OR diet) ADJ3 carbohydrate*))).ab,ti,kf.) AND English.lg. not (exp animals/ not humans/) NOT (editorial/ or letter/)  limit to yr="2010 -Current" | 07.02.20 | 218 | 217 |
| Embase | ('liver surgery'/de OR 'liver resection'/exp OR 'liver disease'/exp/mj/dm_su OR (Hepatectom* OR Hemihepatectom* OR ((Hepatic OR Liver) NEAR/3 (Resection* OR Lobectom* OR surg* OR operation* OR segmentectom*))):ab,ti,kw) AND ('diet restriction'/de OR 'carbohydrate'/de OR 'carbohydrate intake'/de OR 'carbohydrate diet'/exp OR 'carbohydrate loading diet'/exp OR 'low calory diet'/de OR (fasting OR ((preoperative OR pre-operative OR overnight OR before-surgery OR before-operation) NEAR/3 fast*) OR ((low-calor* OR hypocaloric OR reducing) NEAR/3 diet) OR ((preoperative OR pre-operative OR drink* OR beverage* OR loading OR diet) NEAR/3 carbohydrate*)):ab,ti,kw) AND [english]/lim AND [2010-2020]/py NOT ([animals]/lim NOT [humans]/lim) NOT ('conference abstract'/it OR 'conference review'/it OR 'editorial'/it OR 'letter'/it) | 07.02.20 | 189 | 113 |
| Cochrane Library | (Hepatectom* OR Hemihepatectom* OR ((Hepatic OR Liver) NEAR/3 (Resection* OR Lobectom* OR surg* OR operation* OR segmentectom*))):ab,ti,kw AND (fasting OR ((preoperative OR pre-operative OR overnight OR before-surgery OR before-operation) NEAR/3 fast*) OR ((low-calor* OR hypocaloric OR reducing) NEAR/3 diet) OR ((preoperative OR pre-operative OR drink* OR beverage* OR loading OR diet) NEAR/3 carbohydrate*)):ab,ti,kw  Publication Year from 2010 to 2020 / NOT Conference abstract | 07.02.20 | 45 | 23 |
| Total |  |  | 452 | 353 |

| 1. Oral Bowel preparation : item not retained |
| --- |

| 1. Pre-anaesthetic medication | | | | |
| --- | --- | --- | --- | --- |
| Database | Search strategy | Date | References | |
|  |  |  | Found | After deduplication |
| Medline OVID SP | (Hepatectomy/ OR ((exp Liver/su OR exp Liver Diseases/su) NOT Liver transplantation/) OR (Hepatectom* OR Hemihepatectom* OR ((Hepatic OR Liver) ADJ3 (Resection* OR Lobectom* OR surg* OR operation* OR segmentectom*))).ab,ti,kf.) AND (Preanesthetic Medication/ OR ((exp "Anti-Anxiety Agents"/) AND (Preoperative Care/ OR Preoperative Period/ OR (pre-anesthetic OR preanesthetic OR pre-anaesthetic OR preanaesthetic OR preoperative* OR pre-operative OR pre-emptive OR preemptive OR short-acting).ab,ti,kf.)) OR (((pre-anesthetic OR preanesthetic OR pre-anaesthetic OR preanaesthetic OR pre-operative OR preoperative OR pre-emptive OR preemptive OR short-acting) ADJ5 (medication OR drug* OR sedative* OR anxiolytic* OR anxiety)) OR premedication).ab,ti,kf.) AND English.lg. not (exp animals/ not humans/) NOT (editorial/ or letter/)  limit to yr="2010 -Current" | 07.02.20 | 10 | 10 |
| Embase | ('liver surgery'/de OR 'liver resection'/exp OR ('liver disease'/exp/dm_su NOT 'liver transplantation'/exp) OR (Hepatectom* OR Hemihepatectom* OR ((Hepatic OR Liver) NEAR/3 (Resection* OR Lobectom* OR surg* OR operation* OR segmentectom*))):ab,ti,kw) AND ('premedication'/exp OR ('anxiolytic agent'/exp AND ('preoperative period'/exp OR (pre-anesthetic OR preanesthetic OR pre-anaesthetic OR preanaesthetic OR preoperative* OR 'pre operative' OR pre-emptive OR preemptive OR short-acting):ab,ti,kw)) OR (((pre-anesthetic OR preanesthetic OR pre-anaesthetic OR preanaesthetic OR pre-operative OR preoperative OR pre-emptive OR preemptive OR short-acting) NEAR/5 (medication OR drug* OR sedative* OR anxiolytic* OR anxiety)) OR premedication):ab,ti,kw) AND [english]/lim AND [2010-2020]/py NOT ([animals]/lim NOT [humans]/lim) NOT ('conference abstract'/it OR 'conference review'/it OR 'editorial'/it OR 'letter'/it) | 07.02.20 | 80 | 72 |
| Cochrane Library | (Hepatectom* OR Hemihepatectom* OR ((Hepatic OR Liver) NEAR/3 (Resection* OR Lobectom* OR surg* OR operation* OR segmentectom*))):ab,ti,kw AND (((pre-anesthetic OR preanesthetic OR pre-anaesthetic OR preanaesthetic OR pre-operative OR preoperative OR pre-emptive OR preemptive OR short-acting) NEAR/5 (medication OR drug* OR sedative* OR anxiolytic* OR anxiety)) OR premedication):ab,ti,kw  Publication Year from 2010 to 2020 / NOT Conference abstract | 07.02.20 | 25 | 20 |
| Total |  | 07.02.20 | 115 | 102 |

| 1. Anti-thrombotic prophylaxis | | | | |
| --- | --- | --- | --- | --- |
| Database | Search strategy | Date | References | |
|  |  |  | Found | After deduplication |
| Medline OVID SP | (Hepatectomy/ OR ((exp Liver/su OR exp Liver Diseases/su) NOT Liver transplantation/) OR (Hepatectom* OR Hemihepatectom* OR ((Hepatic OR Liver) ADJ3 (Resection* OR Lobectom* OR surg* OR operation* OR segmentectom*))).ab,ti,kf.) AND (exp embolism/pc, th, dt or exp thromboembolism/pc, th, dt or exp thrombosis/pc, th, dt OR exp "Heparin, Low-Molecular-Weight"/ OR (((anti-thrombotic OR antithrombotic) ADJ2 (prophylaxis OR chemoprophylaxis)) OR heparin OR thromboprophylaxis OR ((thromboemboli* OR thrombosis) AND (preventi* OR prophylaxis OR chemoprophylaxis))).ab,ti,kf.) AND English.lg. not (exp animals/ not humans/) NOT (editorial/ or letter/)  limit to yr="2010 -Current" | 07.02.20 | 176 | 173 |
| Embase | ('liver surgery'/de OR 'liver resection'/exp OR ('liver disease'/exp/dm_su NOT 'liver transplantation'/exp) OR (Hepatectom* OR Hemihepatectom* OR ((Hepatic OR Liver) NEAR/3 (Resection* OR Lobectom* OR surg* OR operation* OR segmentectom*))):ab,ti,kw) AND ('thromboembolism'/exp/dm_dt,dm_pc,dm_th OR ('thromboembolism'/exp AND ('prophylaxis'/de OR 'chemoprophylaxis'/de)) OR 'thrombosis prevention'/de OR 'low molecular weight heparin'/exp OR (((anti-thrombotic OR antithrombotic) NEAR/2 prophylaxis) OR heparin OR thromboprophylaxis OR ((thromboemboli* OR thrombosis) AND (preventi* OR prophylaxis OR chemoprophylaxis))):ab,ti,kw) AND [english]/lim AND [2010-2020]/py NOT ([animals]/lim NOT [humans]/lim) NOT ('conference abstract'/it OR 'conference review'/it OR 'editorial'/it OR 'letter'/it) | 07.02.20 | 489 | 388 |
| Cochrane Library | (Hepatectom* OR Hemihepatectom* OR ((Hepatic OR Liver) NEAR/3 (Resection* OR Lobectom* OR surg* OR operation* OR segmentectom*))):ab,ti,kw AND (((anti-thrombotic OR antithrombotic) NEAR/2 prophylaxis) OR heparin OR thromboprophylaxis OR ((thromboemboli* OR thrombosis) AND (preventi* OR prophylaxis OR chemoprophylaxis))):ab,ti,kw  Publication Year from 2010 to 2020 / NOT Conference abstract | 07.02.20 | 27 | 19 |

| 1. Perioperative steroid administration | | | | |
| --- | --- | --- | --- | --- |
| Database | Search strategy | Date | References | |
|  |  |  | Found | After deduplication |
| Medline OVID SP | (Hepatectomy/ OR ((exp Liver/su OR exp Liver Diseases/su) NOT Liver transplantation/) OR (Hepatectom* OR Hemihepatectom* OR ((Hepatic OR Liver) ADJ3 (Resection* OR Lobectom* OR surg* OR operation* OR segmentectom*))).ab,ti,kf.) AND (exp Steroids/ OR (steroid* OR methylprednisolone).ab,ti,kf.) AND (Preoperative Care/ OR Preoperative Period/ OR Perioperative Care/ OR exp Perioperative Period/ OR Perioperative Medicine/ OR exp Perioperative Care/ OR (preoperative* OR pre-operative OR perioperative OR peri-operative OR before-surgery OR before-operation).ab,ti,kf.) AND English.lg. not (exp animals/ not humans/) NOT (editorial/ or letter/)  limit to yr="2010 -Current" | 10.02.20 | 44 | 43 |
| Embase | ('liver surgery'/de OR 'liver resection'/exp OR ('liver disease'/exp/dm_su NOT 'liver transplantation'/exp) OR (Hepatectom* OR Hemihepatectom* OR ((Hepatic OR Liver) NEAR/3 (Resection* OR Lobectom* OR surg* OR operation* OR segmentectom*))):ab,ti,kw) AND ('steroid'/exp OR (steroid* OR methylprednisolone):ab,ti,kw) AND ('preoperative period'/exp OR 'perioperative period'/de OR (preoperative* OR pre-operative OR perioperative OR peri-operative OR before-surgery OR before-operation):ab,ti,kw) AND [english]/lim AND [2010-2020]/py NOT ([animals]/lim NOT [humans]/lim) NOT ('conference abstract'/it OR 'conference review'/it OR 'editorial'/it OR 'letter'/it) | 10.02.20 | 167 | 139 |
| Cochrane Library | (Hepatectom* OR Hemihepatectom* OR ((Hepatic OR Liver) NEAR/3 (Resection* OR Lobectom* OR surg* OR operation* OR segmentectom*))):ab,ti,kw AND (steroid* OR methylprednisolone):ab,ti,kw AND (preoperative* OR pre-operative OR perioperative OR "peri-operative"):ab,ti,kw  Publication Year from 2010 to 2020 | 10.02.20 | 12 | 7 |
| Total |  | 10.02.20 | 223 | 189 |

| 1. Antimicrobial prophylaxis and skin preparation | | | | |
| --- | --- | --- | --- | --- |
| Database | Search strategy | Date | References | |
|  |  |  | Found | After deduplication |
| Medline OVID SP | (Hepatectomy/ OR ((exp Liver/su OR exp Liver Diseases/su) NOT Liver transplantation/) OR (Hepatectom* OR Hemihepatectom* OR ((Hepatic OR Liver) ADJ3 (Resection* OR Lobectom* OR surg* OR operation* OR segmentectom*))).ab,ti,kf.) AND (Antibiotic Prophylaxis/ OR Bacterial Infections/pc OR ((antimicrobial OR antibiotic) ADJ6 (prophyla* OR chemoprophylaxis)).ab,ti,kf. OR Surgical Wound Infection/pc OR (*Anti-Bacterial Agents/tu AND (Preoperative Care/ OR Preoperative Period/ OR Perioperative Care/ OR exp Perioperative Period/ OR Perioperative Medicine/ OR exp Perioperative Care/ OR (preoperative* OR pre-operative OR perioperative OR peri-operative).ab,ti,kf)) OR (Anti-Bacterial Agents/ad, tu AND (Surgical Wound/ OR ("skin preparation" OR incision).ab,ti,kf.)) OR ((skin OR "surgical site") ADJ3 (antisepsis OR infecti* OR disinfection OR alcohol OR chlorhexidine OR povidone-iodine)).ab,ti,kf.) AND English.lg. not (exp animals/ not humans/) NOT (editorial/ or letter/)  limit to yr="2010 -Current" | 11.02.20 | 169 | 166 |
| Embase | ('liver surgery'/de OR 'liver resection'/exp OR ('liver disease'/exp/dm_su NOT 'liver transplantation'/exp) OR (Hepatectom* OR Hemihepatectom* OR ((Hepatic OR Liver) NEAR/3 (Resection* OR Lobectom* OR surg* OR operation* OR segmentectom*))):ab,ti,kw) AND ('antibiotic prophylaxis'/de OR 'bacterial infection'/exp/dm_pc OR 'skin decontamination'/de OR ('antiinfective agent'/exp/mj/dd_dt AND ('preoperative period'/exp OR 'perioperative period'/de OR (perioperative OR 'peri operative' OR preoperative OR 'pre operative'):ab,ti,kw)) OR ((antimicrobial OR antibiotic*) NEAR/6 (prophyla* OR chemoprophylaxis OR perioperative OR "peri operative" OR preoperative OR "pre operative")):ab,ti,kw OR 'surgical infection'/de/dm_pc OR ('antiinfective agent'/exp AND ('skin incision'/de OR 'skin preparation'/de OR ("skin preparation" OR incision):ab,ti,kw)) OR ((skin OR "surgical site") NEAR/3 (antisepsis OR infecti* OR disinfection OR alcohol OR chlorhexidine OR povidone-iodine)):ab,ti,kw) AND [english]/lim AND [2010-2020]/py NOT ([animals]/lim NOT [humans]/lim) NOT ('conference abstract'/it OR 'conference review'/it OR 'editorial'/it OR 'letter'/it) | 11.02.20 | 315 | 180 |
| Cochrane Library | (Hepatectom* OR Hemihepatectom* OR ((Hepatic OR Liver) NEAR/3 (Resection* OR Lobectom* OR surg* OR operation* OR segmentectom*))):ab,ti,kw AND (((antimicrobial OR antibiotic*) NEAR/6 (prophyla* OR chemoprophylaxis OR perioperative OR "peri operative" OR preoperative OR "pre operative")):ab,ti,kw OR ((skin OR "surgical site") NEAR/3 (antisepsis OR infecti* OR disinfection OR alcohol OR chlorhexidine OR "povidone iodine")):ab,ti,kw)  Publication Year from 2010 to 2020 | 11.02.20 | 46 | 24 |
| Total |  | 11.02.20 | 530 | 370 |

| 1. Incision : item not retained |
| --- |

| 1. Minimal invasive surgery | | | | |
| --- | --- | --- | --- | --- |
| Database | Search strategy | Date | References | |
|  |  |  | Found | After deduplication |
| Medline OVID SP | (Hepatectomy/ OR ((exp Liver/su OR exp Liver Diseases/su) NOT Liver transplantation/) OR (Hepatectom* OR Hemihepatectom* OR ((Hepatic OR Liver) ADJ3 (Resection* OR Lobectom* OR surg* OR operation* OR segmentectom*))).ab,ti,kf.) AND (exp Laparoscopy/ OR "Minimally Invasive Surgical Procedures"/ OR "Robotic Surgical Procedures"/ OR ((laparoscop* OR robot* OR "minimal* invasive") ADJ5 (surgery OR Hepatectom* OR Hemihepatectom* OR resection* OR lobectom* OR segmentectom*)).ab,ti,kf.) *AND (exp "Controlled Clinical Trial"/ OR exp "Controlled Clinical Trials as Topic"/ OR "Meta-Analysis"/ OR exp "Meta-Analysis as Topic"/ OR "Systematic Review"/ OR exp guideline/ OR "Cohort Studies"/ OR "Prospective Studies"/ OR "Longitudinal Studies"/ OR (randomized OR randomised OR (systematic ADJ3 review*) OR met-analy* or metanaly*).ab,ti. OR (recommendation* OR guideline).ti)* AND English.lg. not (exp animals/ not humans/) NOT (editorial/ or letter/)  limit to yr="2010 -Current" | 11.02.20 | 491 | 489 |
| Embase | ('liver surgery'/de OR 'liver resection'/exp OR ('liver disease'/exp/dm_su NOT 'liver transplantation'/exp) OR (Hepatectom* OR Hemihepatectom* OR ((Hepatic OR Liver) NEAR/3 (Resection* OR Lobectom* OR surg* OR operation* OR segmentectom*))):ab,ti,kw) AND ('laparoscopy'/exp OR 'robot assisted surgery'/de OR "Minimally Invasive Surgical Procedures"/de OR (laparoscop* OR ((robot* OR "minimal* invasive") NEAR/5 (surgery OR Hepatectom* OR Hemihepatectom* OR resection* OR lobectom* OR segmentectom*))):ab,ti,kw) *AND ('controlled clinical trial'/exp OR 'major clinical study'/exp OR 'meta analysis'/exp OR 'systematic review'/exp OR 'randomized controlled trial (topic)'/exp OR 'systematic review (topic)'/exp OR 'practice guideline'/de OR 'prospective study'/de OR 'cohort analysis'/de OR 'longitudinal study'/de OR (randomized OR randomised OR (systematic NEAR/3 review*) OR met-analy* or metanaly*):ab,ti OR (recommendation* OR guideline):ti)* AND [english]/lim AND [2010-2020]/py NOT ([animals]/lim NOT [humans]/lim) NOT ('conference abstract'/it OR 'conference review'/it OR 'editorial'/it OR 'letter'/it) | 11.02.20 | 1407 | 1013 |
| Cochrane Library | (Hepatectom* OR Hemihepatectom* OR ((Hepatic OR Liver) NEAR/3 (Resection* OR Lobectom* OR surg* OR operation* OR segmentectom*))):ab,ti,kw AND (laparoscop* OR ((robot* OR "minimal invasive" OR "minimally invasive") NEAR/5 (surgery OR Hepatectom* OR Hemihepatectom* OR resection* OR lobectom* OR segmentectom*))):ab,ti,kw  Publication Year from 2010 to 2020 | 11.02.20 | 265 | 160 |
| Total |  | 11.02.20 | 2163 | 1662 |

*Addition of a filter for some specific types of studies*

| 1. Nasogastric intubation | | | | |
| --- | --- | --- | --- | --- |
| Database | Search strategy | Date | References | |
|  |  |  | Found | After deduplication |
| Medline OVID SP | (Hepatectomy/ OR ((exp Liver/su OR exp Liver Diseases/su) NOT Liver transplantation/) OR (Hepatectom* OR Hemihepatectom* OR ((Hepatic OR Liver) ADJ3 (Resection* OR Lobectom* OR surg* OR operation* OR segmentectom*))).ab,ti,kf.) AND ("Intubation, Gastrointestinal"/ OR (nasogastric ADJ3 (tube* OR intuba* OR decompression)).ab,ti,kf.) AND English.lg. not (exp animals/ not humans/) NOT (editorial/ or letter/)  limit to yr="2010 -Current" | 11.02.20 | 20 | 20 |
| Embase | ('liver surgery'/de OR 'liver resection'/exp OR ('liver disease'/exp/dm_su NOT 'liver transplantation'/exp) OR (Hepatectom* OR Hemihepatectom* OR ((Hepatic OR Liver) NEAR/3 (Resection* OR Lobectom* OR surg* OR operation* OR segmentectom*))):ab,ti,kw) AND ('nasogastric tube'/exp OR 'stomach intubation'/de OR (nasogastric NEAR/3 (tube* OR intuba* OR decompression)):ab,ti,kw) AND [english]/lim AND [2010-2020]/py NOT ([animals]/lim NOT [humans]/lim) NOT ('conference abstract'/it OR 'conference review'/it OR 'editorial'/it OR 'letter'/it) | 11.02.20 | 50 | 35 |
| Cochrane Library | (Hepatectom* OR Hemihepatectom* OR ((Hepatic OR Liver) NEAR/3 (Resection* OR Lobectom* OR surg* OR operation* OR segmentectom*))):ab,ti,kw AND (nasogastric NEAR/3 (tube* OR intuba* OR decompression)):ab,ti,kw  Publication Year from 2010 to 2020 | 11.02.20 | 12 | 8 |
| Total |  | 11.02.20 | 82 | 63 |

| 1. Prophylactic abdominal drainage | | | | |
| --- | --- | --- | --- | --- |
| Database | Search strategy | Date | References | |
|  |  |  | Found | After deduplication |
| Medline OVID SP | (Hepatectomy/ OR ((exp Liver/su OR exp Liver Diseases/su) NOT Liver transplantation/) OR (Hepatectom* OR Hemihepatectom* OR ((Hepatic OR Liver) ADJ3 (Resection* OR Lobectom* OR surg* OR operation* OR segmentectom*))).ab,ti,kf.) AND ((("Drainage"/ OR "Suction"/) AND (exp "Controlled Clinical Trial"/ OR exp "Controlled Clinical Trials as Topic"/ OR "Meta-Analysis"/ OR exp "Meta-Analysis as Topic"/ OR "Systematic Review"/ OR exp guideline/ OR (randomized OR randomised OR (systematic ADJ3 review*) OR met-analy* or metanaly*).ab,ti,kf OR (recommendation* OR guideline).ti)) OR (("Drainage"/ OR "Suction"/ OR drain*.ab,ti,kf.) AND (exp Abscess/pc OR Digestive System Fistula/pc OR Biliary Fistula/pc OR (prophyla* OR prevent*).ab,ti,kf.)) OR ((intraoperativ* OR intra-operative OR operative OR peroperative OR primary OR routine) ADJ3 drain*).ab,ti,kf.) AND English.lg. not (exp animals/ not humans/) NOT (editorial/ or letter/)  limit to yr="2010 -Current" | 11.02.20 | 182 | 178 |
| Embase | ('liver surgery'/de OR 'liver resection'/exp OR ('liver disease'/exp/dm_su NOT 'liver transplantation'/exp) OR (Hepatectom* OR Hemihepatectom* OR ((Hepatic OR Liver) NEAR/3 (Resection* OR Lobectom* OR surg* OR operation* OR segmentectom*))):ab,ti,kw) AND ((('surgical drainage'/de OR 'abdominal drainage'/exp OR 'abscess drainage'/de OR 'closed drainage'/exp OR 'suction drainage'/de OR 'wound drainage'/de) AND ('controlled clinical trial'/exp OR 'major clinical study'/exp OR 'meta analysis'/exp OR 'systematic review'/exp OR 'randomized controlled trial (topic)'/exp OR 'systematic review (topic)'/exp OR 'practice guideline'/de OR (randomized OR randomised OR (systematic NEAR/3 review*) OR met-analy* or metanaly*):ab,ti OR (guideline* OR recommendation*):ti)) OR (('surgical drainage'/de OR 'abdominal drainage'/exp OR 'abscess drainage'/de OR 'closed drainage'/exp OR 'suction drainage'/de OR 'wound drainage'/de OR drain*:ab,ti,kw) AND ('abscess'/exp/dm_pc OR 'digestive system fistula'/exp/dm_pc OR 'biloma'/exp/dm_pc OR (prophyla* OR prevent*):ab,ti,kw)) OR ((intraoperativ* OR intra-operative OR operative OR peroperative OR primary OR routine) NEAR/3 drain*):ab,ti,kw) AND [english]/lim AND [2010-2020]/py NOT ([animals]/lim NOT [humans]/lim) NOT ('conference abstract'/it OR 'conference review'/it OR 'editorial'/it OR 'letter'/it) | 11.02.20 | 262 | 135 |
| Cochrane Library | (Hepatectom* OR Hemihepatectom* OR ((Hepatic OR Liver) NEAR/3 (Resection* OR Lobectom* OR surg* OR operation* OR segmentectom*))):ab,ti,kw AND ((drain* AND (prophyla* OR prevent*)):ab,ti,kw OR ((intraoperativ* OR intra-operative OR operative OR peroperative OR primary OR routine) NEAR/3 drain*):ab,ti,kw)  Publication Year from 2010 to 2020 | 11.02.20 | 46 | 25 |
| Total |  | 11.02.20 | 490 | 338 |

| 1. Preventing intraoperative Hypothermia | | | | |
| --- | --- | --- | --- | --- |
| Database | Search strategy | Date | References | |
|  |  |  | Found | After deduplication |
| Medline OVID SP | (Hepatectomy/ OR ((exp Liver/su OR exp Liver Diseases/su) NOT Liver transplantation/) OR (Hepatectom* OR Hemihepatectom* OR ((Hepatic OR Liver) ADJ3 (Resection* OR Lobectom* OR surg* OR operation* OR segmentectom*))).ab,ti,kf.) AND (Hypothermia/ OR Hypothermia, Induced/ OR exp Body Temperature/ OR Body Temperature Regulation/ OR Rewarming/ OR (hypothermia OR warming OR heating OR ((warm* OR heat*) ADJ3 (garment* OR mattress* or air or water)) OR normothermia OR thermoregulation OR ((temperature OR heat) ADJ3 (control* OR measure* OR monitor* OR intraoperative OR body OR operative OR loss))).ab,ti,kf.) AND English.lg. NOT (exp animals/ not humans/) NOT (editorial/ or letter/)  limit to yr="2010 -Current" | 13.02.20 | 128 | 128 |
| Embase | ('liver surgery'/de OR 'liver resection'/exp OR ('liver disease'/exp/dm_su NOT 'liver transplantation'/exp) OR (Hepatectom* OR Hemihepatectom* OR ((Hepatic OR Liver) NEAR/3 (Resection* OR Lobectom* OR surg* OR operation* OR segmentectom*))):ab,ti,kw) AND ('hypothermia'/exp OR 'body temperature'/exp OR 'induced hypothermia'/exp OR 'body temperature monitoring'/de OR 'temperature measurement'/exp OR 'warming'/de OR 'thermal regulating system'/exp OR 'thermoregulation'/de OR (hypothermia OR warming OR heating OR ((warm* OR heat*) NEXT/3 (garment* OR mattress* OR air OR water)) OR normothermia OR thermoregulation OR ((temperature OR heat) NEAR/3 (control* OR measure* OR monitor* OR intraoperative OR body OR operative OR loss))):ab,ti,kw) AND [english]/lim AND [2010-2020]/py NOT ([animals]/lim NOT [humans]/lim) NOT ('conference abstract'/it OR 'conference review'/it OR 'editorial'/it OR 'letter'/it) | 13.02.20 | 176 | 116 |
| Cochrane Library | (Hepatectom* OR Hemihepatectom* OR ((Hepatic OR Liver) NEAR/3 (Resection* OR Lobectom* OR surg* OR operation* OR segmentectom*))):ab,ti,kw AND (hypothermia OR warming OR heating OR ((warm* OR heat*) NEXT/3 (garment* OR mattress* OR air OR water)) OR normothermia OR thermoregulation OR ((temperature OR heat) NEAR/3 (control* OR measure* OR monitor* OR intraoperative OR body OR operative OR loss))):ab,ti,kw  Publication Year from 2010 to 2020 | 13.02.20 | 28 | 18 |
| Total |  | 13.02.20 | 332 | 262 |

| 1. Postoperative nutrition and early oral intake | | | | |
| --- | --- | --- | --- | --- |
| Database | Search strategy | Date | References | |
|  |  |  | Found | After deduplication |
| Medline OVID SP | (Hepatectomy/ OR ((exp Liver/su OR exp Liver Diseases/su) NOT Liver transplantation/) OR (Hepatectom* OR Hemihepatectom* OR ((Hepatic OR Liver) ADJ3 (Resection* OR Lobectom* OR surg* OR operation* OR segmentectom*))).ab,ti,kf.) AND (((exp Nutrition Therapy/ OR exp Feeding Methods/ OR exp Food/ OR exp Diet/ OR exp Eating/ OR (nutrition OR "nutritional support" OR supplement* OR food OR feeding OR diet* OR eat*).ab,ti,kf.) AND (exp "Postoperative Period"/ OR exp "Postoperative Complications"/ OR "Postoperative Care"/ OR Perioperative Period/ OR "Perioperative Care"/ OR Perioperative Medicine/ OR (postoperative OR "post operative" OR perioperative OR "peri operative" OR ((after OR follow*) ADJ3 (hepatectom* OR hemihepatectom* OR resection* OR lobectom* OR surgery OR operation* OR segmentectom*))).ab,ti,kf.)) OR ((normal OR early) ADJ3 (intake OR diet OR feeding OR nutrition OR food OR eat*)).ab,ti,kf.) AND English.lg. not (exp animals/ not humans/) NOT (editorial/ or letter/)  limit to yr="2010 -Current" | 13.02.20 | 376 | 375 |
| Embase | ('liver surgery'/de OR 'liver resection'/exp OR ('liver disease'/exp/dm_su NOT 'liver transplantation'/exp) OR (Hepatectom* OR Hemihepatectom* OR ((Hepatic OR Liver) NEAR/3 (Resection* OR Lobectom* OR surgery OR operation* OR segmentectom*))):ab,ti,kw) AND ((('artificial feeding'/exp OR 'nutrition'/de OR 'diet'/exp OR 'dietary intake'/exp OR 'food intake'/exp OR 'meal'/exp OR 'diet therapy'/exp OR (nutrition OR "nutritional support" OR supplement* OR food of feeding OR diet* OR eat*):ab,ti,kw) AND ('postoperative period'/de OR 'postoperative care'/de OR 'postoperative complication'/de OR 'perioperative period'/exp OR (postoperative OR "post operative" OR perioperative OR "peri operative" OR ((after OR follow*) NEAR/3 (hepatectom* OR hemihepatectom* OR resection* OR lobectom* OR surgery OR operation* OR segmentectom*))):ab,ti,kw)) OR ((normal OR early) NEAR/3 (intake OR diet OR feeding OR nutrition OR food OR eat*)):ab,ti,kw) AND [english]/lim AND [2010-2020]/py NOT ([animals]/lim NOT [humans]/lim) NOT ('conference abstract'/it OR 'conference review'/it OR 'editorial'/it OR 'letter'/it) | 13.02.20 | 410 | 247 |
| Cochrane Library | (Hepatectom* OR Hemihepatectom* OR ((Hepatic OR Liver) NEAR/3 (Resection* OR Lobectom* OR surg* OR operation* OR segmentectom*))):ab,ti,kw AND ((nutrition OR "nutritional support" OR supplement* OR food OR feeding OR diet* OR eat*) AND (postoperative OR "post operative" OR perioperative OR "peri operative" OR ((after OR follow*) NEAR/3 (hepatectom* OR hemihepatectom* OR resection* OR lobectom* OR surgery OR operation* OR segmentectom*)))):ab,ti,kw  Publication Year from 2010 to 2020 | 13.02.20 | 144 | 90 |
| Total |  | 13.02.20 | 930 | 712 |

| 1. Postoperative Glycaemic control | | | | |
| --- | --- | --- | --- | --- |
| Database | Search strategy | Date | References | |
|  |  |  | Found | After deduplication |
| Medline OVID SP | (Hepatectomy/ OR ((exp Liver/su OR exp Liver Diseases/su) NOT Liver transplantation/) OR (Hepatectom* OR Hemihepatectom* OR ((Hepatic OR Liver) ADJ3 (Resection* OR Lobectom* OR surg* OR operation* OR segmentectom*))).ab,ti,kf.) AND (Hyperglycemia/ OR Insulin Resistance/ OR exp insulins/ OR (hyperglycemia OR hyperglycaemia OR ((glycaemic OR glycemic OR glucose) ADJ3 (control* OR management OR level* OR plasma OR blood OR monitor*)) OR (insulin ADJ3 resistance) OR (Insulin ADJ3 (therapy OR administ* OR treat*))).ab,ti,kf.) AND English.lg. not (exp animals/ not humans/) NOT (editorial/ or letter/)  limit to yr="2010 -Current" | 02.04.20 | 167 | 166 |
| Embase | ('liver surgery'/de OR 'liver resection'/exp OR ('liver disease'/exp/dm_su NOT 'liver transplantation'/exp) OR (Hepatectom* OR Hemihepatectom* OR ((Hepatic OR Liver) NEAR/3 (Resection* OR Lobectom* OR surg* OR operation* OR segmentectom*))):ab,ti,kw) AND ('hyperglycemia'/de OR 'glycemic control'/de OR 'blood glucose monitoring'/de OR 'glucose blood level'/de OR 'insulin resistance'/de OR 'insulin derivative'/exp OR 'insulin treatment'/exp OR (hyperglycemia OR hyperglycaemia OR ((glycaemic OR glycemic OR glucose) NEAR/3 (control* OR management OR level* OR plasma OR blood OR monitor*)) OR (insulin NEAR/3 resistance) OR (Insulin NEAR/3 (therapy OR administ* OR treat*))):ab,ti,kw) AND [english]/lim AND [2010-2020]/py NOT ([animals]/lim NOT [humans]/lim) NOT ('conference abstract'/it OR 'conference review'/it OR 'editorial'/it OR 'letter'/it) | 02.04.20 | 498 | 385 |
| Cochrane Library | (Hepatectom* OR Hemihepatectom* OR ((Hepatic OR Liver) NEAR/3 (Resection* OR Lobectom* OR surg* OR operation* OR segmentectom*))):ab,ti,kw AND (hyperglycemia OR hyperglycaemia OR ((glycaemic or glycemic OR glucose) NEAR/3 (control* OR management OR level* OR plasma OR blood OR monitor*)) OR (insulin NEAR/3 resistance) OR (Insulin NEAR/3 (therapy OR administ* OR treat*))):ab,ti,kw  Publication Year from 2010 to 2020 | 02.04.20 | 67 | 31 |
| Total |  |  | 732 | 582 |

| 1. Delayed gastric emptying | | | | |
| --- | --- | --- | --- | --- |
| Database | Search strategy | Date | References | |
|  |  |  | Found | After deduplication |
| Medline OVID SP | (Hepatectomy/ OR ((exp Liver/su OR exp Liver Diseases/su) NOT Liver transplantation/) OR (Hepatectom* OR Hemihepatectom* OR ((Hepatic OR Liver) ADJ3 (Resection* OR Lobectom* OR surg* OR operation* OR segmentectom*))).ab,ti,kf.) AND (Gastric Emptying/ OR (delay* ADJ3 (stomach OR gastric) ADJ3 empty*).ab,ti,kf.) AND English.lg. not (exp animals/ not humans/) NOT (editorial/ or letter/)  limit to yr="2010 -Current" | 31.03.20 | 12 | 12 |
| Embase | ('liver surgery'/de OR 'liver resection'/exp OR ('liver disease'/exp/dm_su NOT 'liver transplantation'/exp) OR (Hepatectom* OR Hemihepatectom* OR ((Hepatic OR Liver) NEAR/3 (Resection* OR Lobectom* OR surg* OR operation* OR segmentectom*))):ab,ti,kw) AND ('stomach emptying'/exp OR (delay* NEAR/3 (stomach OR gastric) NEAR/3 empty*):ab,ti,kw) AND [english]/lim AND [2010-2020]/py NOT ([animals]/lim NOT [humans]/lim) NOT ('conference abstract'/it OR 'conference review'/it OR 'editorial'/it OR 'letter'/it) | 31.03.20 | 38 | 30 |
| Cochrane Library | (Hepatectom* OR Hemihepatectom* OR ((Hepatic OR Liver) NEAR/3 (Resection* OR Lobectom* OR surg* OR operation* OR segmentectom*))):ab,ti,kw AND (delay* NEAR/3 (stomach OR gastric) NEAR/3 empty*):ab,ti,kw  Publication Year from 2010 to 2020 | 31.03.20 | 5 | 4 |
| Total |  | 31.03.20 | 55 | 46 |

| 1. Stimulation of bowel movement | | | | |
| --- | --- | --- | --- | --- |
| Database | Search strategy | Date | References | |
|  |  |  | Found | After deduplication |
| Medline OVID SP | (Hepatectomy/ OR ((exp Liver/su OR exp Liver Diseases/su) NOT Liver transplantation/) OR (Hepatectom* OR Hemihepatectom* OR ((Hepatic OR Liver) ADJ3 (Resection* OR Lobectom* OR surg* OR operation* OR segmentectom*))).ab,ti,kf.) AND (Exp "Gastrointestinal Transit"/ OR "Gastrointestinal Motility"/ OR "Defecation"/ OR exp "Constipation"/ OR "Intestinal Obstruction"/ OR exp "Intestinal Pseudo-Obstruction"/ OR laxatives/ OR (((Bowel OR gastrointestinal OR intestin* OR gut OR colonic) ADJ3 (transit OR movement OR motility OR dysmotility OR activity OR paralysis)) OR (bowel ADJ3 function) OR defecation OR constipation OR laxative* OR ((postoperative OR paralytic) ADJ1 ileus)).ab,ti,kf.) AND (exp "Postoperative Period"/ OR exp "Postoperative Complications"/ OR "Postoperative Care"/ OR Perioperative Period/ OR "Perioperative Care"/ OR Perioperative Medicine/ OR (postoperative OR "post operative" OR perioperative OR "peri operative" OR ((after OR follow*) ADJ3 (hepatectom* OR hemihepatectom* OR resection* OR lobectom* OR surgery OR operation* OR segmentectom*))).ab,ti,kf.) AND English.lg. not (exp animals/ not humans/) NOT (editorial/ or letter/)  limit to yr="2010 -Current" | 02.04.20 | 68 | 68 |
| Embase | ('liver surgery'/de OR 'liver resection'/exp OR ('liver disease'/exp/dm_su NOT 'liver transplantation'/exp) OR (Hepatectom* OR Hemihepatectom* OR ((Hepatic OR Liver) NEAR/3 (Resection* OR Lobectom* OR surg* OR operation* OR segmentectom*))):ab,ti,kw) AND ('gastrointestinal motility'/de OR 'intestine motility'/exp OR 'gastrointestinal tract function'/exp OR 'gastrointestinal transit'/exp OR 'defecation'/de OR 'constipation'/de OR 'intestinal dysmotility'/de OR 'intestine obstruction'/de OR 'postoperative ileus'/de OR 'paralytic ileus'/de OR 'laxative'/exp OR (((Bowel OR gastrointestinal OR intestin* OR gut OR colonic) NEAR/3 (transit OR movement OR motility OR dysmotility OR activity OR paralysis)) OR (bowel NEAR/3 function) OR defecation OR constipation OR laxative* OR ((postoperative OR paralytic) NEXT/1 ileus)):ab,ti,kw) AND ('postoperative period'/de OR 'postoperative care'/de OR 'postoperative complication'/de OR 'perioperative period'/exp OR (postoperative OR "post operative" OR perioperative OR "peri operative" OR ((after OR follow*) NEAR/3 (hepatectom* OR hemihepatectom* OR resection* OR lobectom* OR surgery OR operation* OR segmentectom*))):ab,ti,kw) AND [english]/lim AND [2010-2020]/py NOT ([animals]/lim NOT [humans]/lim) NOT ('conference abstract'/it OR 'conference review'/it OR 'editorial'/it OR 'letter'/it) | 02.04.20 | 346 | 297 |
| Cochrane Library | (Hepatectom* OR Hemihepatectom* OR ((Hepatic OR Liver) NEAR/3 (Resection* OR Lobectom* OR surg* OR operation* OR segmentectom*))):ab,ti,kw AND (((Bowel OR gastrointestinal OR intestin* OR gut OR colonic) NEAR/3 (transit OR movement OR motility OR dysmotility OR activity OR paralysis)) OR (bowel NEAR/3 function) OR defecation OR constipation OR laxative* OR ((postoperative OR paralytic) NEXT/1 ileus)):ab,ti,kw AND (postoperative OR "post operative" OR perioperative OR "peri operative" OR ((after OR follow*) NEAR/3 (hepatectom* OR hemihepatectom* OR resection* OR lobectom* OR surgery OR operation* OR segmentectom*))):ab,ti,kw  Publication Year from 2010 to 2020 | 02.04.20 | 68 | 41 |
| Total |  |  | 482 | 406 |
| 1. Early Mobilisation | | | | |
| Database | Search strategy | Date | References | |
|  |  |  | Found | After deduplication |
| Medline OVID SP | (Hepatectomy/ OR ((exp Liver/su OR exp Liver Diseases/su) NOT Liver transplantation/) OR (Hepatectom* OR Hemihepatectom* OR ((Hepatic OR Liver) ADJ3 (Resection* OR Lobectom* OR surg* OR operation* OR segmentectom*))).ab,ti,kf.) AND ("Early Ambulation"/ OR (mobilisation OR mobilization OR ambulation).ab,ti,kf.) AND English.lg. not (exp animals/ not humans/) NOT (editorial/ or letter/)  limit to yr="2010 -Current" | 31.03.20 | 199 | 197 |
| Embase | ('liver surgery'/de OR 'liver resection'/exp OR ('liver disease'/exp/dm_su NOT 'liver transplantation'/exp) OR (Hepatectom* OR Hemihepatectom* OR ((Hepatic OR Liver) NEAR/3 (Resection* OR Lobectom* OR surg* OR operation* OR segmentectom*))):ab,ti,kw) AND ('mobilization'/de OR (mobilisation OR mobilization OR ambulation):ab,ti,kw) AND [english]/lim AND [2010-2020]/py NOT ([animals]/lim NOT [humans]/lim) NOT ('conference abstract'/it OR 'conference review'/it OR 'editorial'/it OR 'letter'/it) | 31.03.20 | 223 | 45 |
| Cochrane Library | (Hepatectom* OR Hemihepatectom* OR ((Hepatic OR Liver) NEAR/3 (Resection* OR Lobectom* OR surg* OR operation* OR segmentectom*))):ab,ti,kw AND (mobilisation OR mobilization OR ambulation):ab,ti,kw  Publication Year from 2010 to 2020 | 31.03.20 | 51 | 33 |
| Total |  | 31.03.20 | 473 | 275 |

| 1. Analgesia | | | | |
| --- | --- | --- | --- | --- |
| Database | Search strategy | Date | References | |
|  |  |  | Found | After deduplication |
| Medline OVID SP | (Hepatectomy/ OR ((exp Liver/su OR exp Liver Diseases/su) NOT Liver transplantation/) OR (Hepatectom* OR Hemihepatectom* OR ((Hepatic OR Liver) ADJ3 (Resection* OR Lobectom* OR surg* OR operation* OR segmentectom*))).ab,ti,kf.) AND (exp Analgesia/ OR exp Anesthetics, Local/ OR exp Analgesics/tu OR ((anesthetic* OR anaesthetic*) AND ((wound ADJ3 infusion) OR (local ADJ3 infiltration))).ab,ti,kf. OR (wound ADJ3 catheter*).ab,ti,kf. OR (analgesia OR ((epidural OR peridural OR intrathecal) ADJ5 (opioid* OR opiate* OR morphine OR fentanyl))).ab,ti,kf.) AND English.lg. not (exp animals/ not humans/) NOT (editorial/ or letter/)  limit to yr="2010 -Current" | 02.04.20 | 203 | 203 |
| Embase | ('liver surgery'/de OR 'liver resection'/exp OR ('liver disease'/exp/dm_su NOT 'liver transplantation'/exp) OR (Hepatectom* OR Hemihepatectom* OR ((Hepatic OR Liver) NEAR/3 (Resection* OR Lobectom* OR surg* OR operation* OR segmentectom*))):ab,ti,kw) AND ('analgesia'/exp OR 'analgesic agent'/exp/dd_dt OR 'local anesthetic agent'/exp OR 'local infiltration analgesia'/de OR 'analgesic infusion pump'/exp OR ((anesthetic* OR anaesthetic*) AND ((wound NEAR/3 infusion) OR (local NEAR/3 infiltration))):ab,ti,kw OR (wound NEAR/3 catheter*):ab,ti,kw OR (analgesia OR ((epidural OR peridural OR intrathecal) NEAR/5 (opioid* OR opiate* OR morphine OR fentanyl))):ab,ti,kw) AND [english]/lim AND [2010-2020]/py NOT ([animals]/lim NOT [humans]/lim) NOT ('conference abstract'/it OR 'conference review'/it OR 'editorial'/it OR 'letter'/it) | 02.04.20 | 546 | 383 |
| Cochrane Library | (Hepatectom* OR Hemihepatectom* OR ((Hepatic OR Liver) NEAR/3 (Resection* OR Lobectom* OR surg* OR operation* OR segmentectom*))):ab,ti,kw AND (((anesthetic* OR anaesthetic*) AND ((wound NEAR/3 infusion) OR (local NEAR/3 infiltration))):ab,ti,kw OR (wound NEAR/3 catheter*):ab,ti,kw OR (analgesia OR analgesic* OR "local anesthetic*" OR "local anaesthetic*" OR ((epidural OR peridural OR intrathecal) NEAR/5 (opioid* OR opiate* OR morphine OR fentanyl))):ab,ti,kw)  Publication Year from 2010 to 2020 | 02.04.20 | 172 | 103 |
| Total |  |  | 921 | 689 |

| 1. Postoperative nausea and vomiting (PONV) | | | | |
| --- | --- | --- | --- | --- |
| Database | Search strategy | Date | References | |
|  |  |  | Found | After deduplication |
| Medline OVID SP | (Hepatectomy/ OR ((exp Liver/su OR exp Liver Diseases/su) NOT Liver transplantation/) OR (Hepatectom* OR Hemihepatectom* OR ((Hepatic OR Liver) ADJ3 (Resection* OR Lobectom* OR surg* OR operation* OR segmentectom*))).ab,ti,kf.) AND ("Postoperative Nausea and Vomiting"/ OR ((postoperative ADJ5 (nausea OR vomiting)) OR PONV).ab,ti,kf.) AND English.lg. not (exp animals/ not humans/) NOT (editorial/ or letter/)  limit to yr="2010 -Current" | 31.03.20 | 23 | 23 |
| Embase | ('liver surgery'/de OR 'liver resection'/exp OR ('liver disease'/exp/dm_su NOT 'liver transplantation'/exp) OR (Hepatectom* OR Hemihepatectom* OR ((Hepatic OR Liver) NEAR/3 (Resection* OR Lobectom* OR surg* OR operation* OR segmentectom*))):ab,ti,kw) AND ('postoperative nausea and vomiting'/exp OR 'opioid induced emesis'/de OR (((postoperative OR post-operative) NEAR/5 (nausea* OR vomiting)) OR PONV):ab,ti,kw) AND [english]/lim AND [2010-2020]/py NOT ([animals]/lim NOT [humans]/lim) NOT ('conference abstract'/it OR 'conference review'/it OR 'editorial'/it OR 'letter'/it) | 31.03.20 | 74 | 57 |
| Cochrane Library | (Hepatectom* OR Hemihepatectom* OR ((Hepatic OR Liver) NEAR/3 (Resection* OR Lobectom* OR surg* OR operation* OR segmentectom*))):ab,ti,kw AND (((postoperative OR post-operative) NEAR/5 (nausea* OR vomiting)) OR PONV):ab,ti,kw  Publication Year from 2010 to 2020 | 31.03.20 | 33 | 15 |
| Total |  | 31.03.20 | 130 | 95 |

| 1. Fluid management | | | | |
| --- | --- | --- | --- | --- |
| Database | Search strategy | Date | References | |
|  |  |  | Found | After deduplication |
| Medline OVID SP | (Hepatectomy/ OR ((exp Liver/su OR exp Liver Diseases/su) NOT Liver transplantation/) OR (Hepatectom* OR Hemihepatectom* OR ((Hepatic OR Liver) ADJ3 (Resection* OR Lobectom* OR surg* OR operation* OR segmentectom*))).ab,ti,kf.) AND (Fluid Therapy/ OR Rehydration Solutions/ OR "Hemodynamic Monitoring"/ OR Hemodynamics/ OR "Water-Electrolyte Balance"/ OR "Water-Electrolyte Imbalance"/ OR exp Isotonic Solutions/ OR Central Venous Pressure/ OR ((Fluid* ADJ3 (administration OR volume OR balance OR imbalance OR therapy OR treatment OR management OR infusion OR responsiveness OR replacement OR bolus)) OR "intravascular volume" OR "central venous pressure" OR "stroke volume variation" OR "hemodynamic monitoring" OR crystalloid* OR "normal saline").ab,ti,kw.) AND English.lg. not (exp animals/ not humans/) NOT (editorial/ or letter/)  limit to yr="2010 -Current" | 06.04.20 | 331 | 330 |
| Embase | ('liver surgery'/de OR 'liver resection'/exp OR ('liver disease'/exp/dm_su NOT 'liver transplantation'/exp) OR (Hepatectom* OR Hemihepatectom* OR ((Hepatic OR Liver) NEAR/3 (Resection* OR Lobectom* OR surg* OR operation* OR segmentectom*))):ab,ti,kw) AND ('fluid therapy'/de OR 'rehydration'/de OR 'oral rehydration therapy'/de OR 'hemodynamic monitoring'/exp OR 'hemodynamic parameters'/de OR 'hemodynamics'/de OR 'fluid balance'/de OR 'infusion fluid'/exp OR 'central venous pressure'/de OR 'crystalloid'/de OR ((Fluid* NEAR/3 (administration OR volume OR balance OR imbalance OR therapy OR treatment OR management OR infusion OR responsiveness OR replacement OR bolus)) OR "intravascular volume" OR "central venous pressure" OR "stroke volume variation" OR "hemodynamic monitoring" OR crystalloid* OR "normal saline"):ab,ti,kw) AND [english]/lim AND [2010-2020]/py NOT ([animals]/lim NOT [humans]/lim) NOT ('conference abstract'/it OR 'conference review'/it OR 'editorial'/it OR 'letter'/it) | 06.04.20 | 657 | 427 |
| Cochrane Library | (Hepatectom* OR Hemihepatectom* OR ((Hepatic OR Liver) NEAR/3 (Resection* OR Lobectom* OR surg* OR operation* OR segmentectom*))):ab,ti,kw AND ((Fluid* NEAR/3 (administration OR volume OR balance OR imbalance OR therapy OR treatment OR management OR infusion OR responsiveness OR replacement OR bolus)) OR "intravascular volume" OR "central venous pressure" OR "stroke volume variation" OR "hemodynamic monitoring" OR crystalloid* OR "normal saline"):ab,ti,kw  Publication Year from 2010 to 2020 | 06.04.20 | 157 | 90 |
| Total |  |  | 1145 | 847 |

| 1. Audit | | | | |
| --- | --- | --- | --- | --- |
| Database | Search strategy | Date | References | |
|  |  |  | Found | After deduplication |
| Medline OVID SP | (Hepatectomy/ OR ((exp Liver/su OR exp Liver Diseases/su) NOT Liver transplantation/) OR (Hepatectom* OR Hemihepatectom* OR ((Hepatic OR Liver) ADJ3 (Resection* OR Lobectom* OR surg* OR operation* OR segmentectom*))).ab,ti,kf.) AND (exp "Clinical Audit"/ OR (audit OR audits).ab,ti,kf.) AND English.lg. not (exp animals/ not humans/) NOT (editorial/ or letter/)  limit to yr="2010 -Current" | 13.02.20 | 32 | 32 |
| Embase | ('liver surgery'/de OR 'liver resection'/exp OR ('liver disease'/exp/dm_su NOT 'liver transplantation'/exp) OR (Hepatectom* OR Hemihepatectom* OR ((Hepatic OR Liver) NEAR/3 (Resection* OR Lobectom* OR surg* OR operation* OR segmentectom*))):ab,ti,kw) AND ('clinical audit'/de OR (audit OR audits):ab,ti,kw) AND [english]/lim AND [2010-2020]/py NOT ([animals]/lim NOT [humans]/lim) NOT ('conference abstract'/it OR 'conference review'/it OR 'editorial'/it OR 'letter'/it) | 13.02.20 | 53 | 23 |
| Cochrane Library | (Hepatectom* OR Hemihepatectom* OR ((Hepatic OR Liver) NEAR/3 (Resection* OR Lobectom* OR surg* OR operation* OR segmentectom*))):ab,ti,kw AND (audit OR audits):ab,ti,kw  Publication Year from 2010 to 2020 | 13.02.20 | 1 | 1 |
| Total |  | 13.02.20 | 86 | 56 |
